# Integrative spatial multi-omics reveal niche-specific inflammatory signaling and differentiation hierarchies in AML

**DOI:** 10.1016/j.isci.2025.114289

**Published:** 2025-11-29

**Authors:** Enes Dasdemir, Ivo Veletic, Christopher P. Ly, Andres E. Quesada, Christopher D. Pacheco, Fatima Z. Jelloul, Pamella Borges, Sreyashi Basu, Sonali Jindal, Zhiqiang Wang, Alexander Lazar, Khalida M. Wani, Dinler A. Antunes, Patrick K. Reville, Preethi H. Gunaratne, Robert J. Tower, Padmanee Sharma, Hussein A. Abbas

**Affiliations:** 1 Department of Leukemia, Division of Cancer Medicine, The University of Texas MD Anderson Cancer Center, Houston, TX, USA; 2 Department of Biology and Biochemistry, University of Houston, Houston, TX, USA; 3 Department of Hematopathology, Division of Cancer Medicine, The University of Texas MD Anderson Cancer Center, Houston, TX, USA; 4 Immunotherapy Platform, James P. Allison Institute, The University of Texas MD Anderson Cancer Center, Houston, TX, USA; 5 Department of Pathology, Division of Pathology/Lab Medicine, The University of Texas MD Anderson Cancer Center, Houston, TX, USA; 6 Department of Genitourinary Medical Oncology, Division of Cancer Medicine, The University of Texas MD Anderson Cancer Center, Houston, TX, USA; 7 Department of Surgery, University of Texas Southwestern Medical Center, Dallas, TX, USA; 8 Department of Genomic Medicine, Division of Cancer Medicine, The University of Texas MD Anderson Cancer Center, Houston, TX, USA

**Keywords:** Components of the immune system, Proteomics, Transcriptomics

## Abstract

Acute myeloid leukemia (AML) is a clonal disorder characterized by immature blasts and arrested differentiation that primarily affects the bone marrow (BM) and occasionally presents as extramedullary (EM) disease. EM manifestations highlight AML’s adaptability to distinct microenvironments, which we examined using spatial analyses of medullary and EM tissues. We describe a workflow for Visium-based spatial transcriptomics in medullary and EM AML, revealing insights into cell-cell communication and the spatial organization of AML hierarchies. In BM, monocytes and granulocyte-monocyte progenitors colocalized with leukemic populations, sharing molecular signatures with those in EM sample. CXCL12-CXCR4-mediated communication correlated with PI3K/AKT/mTOR signaling in inflammatory niches. *Trans*-differentiation signals concentrated in AML-infiltrated regions; committed-like AML cells resided in inflammatory niches distant from trabeculae, while primitive-like cells localized near the endosteal niche. GeoMX digital spatial profiling and Opal multiplex fluorescent immunohistochemistry provided orthogonal validation. Overall, our study offers a valuable multimodal resource for exploring AML spatial biology with potential applications in other BM malignancies.

## Highlights

• Spatial multi-omics maps AML states in bone marrow and extramedullary sites
• Primitive AML cells localize near bone; committed-like cells in inflamed regions
• Inflammation drives AML niche remodeling and T cell dysfunction
• CXCL12-CXCR4 signaling axis links AML cells with their microenvironment

## Graphical abstract

**Figure:**
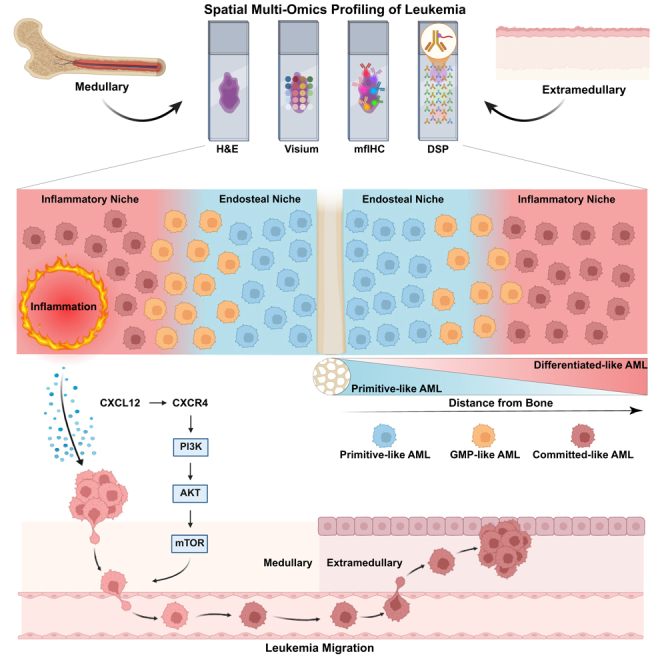

## Introduction

Acute myeloid leukemia (AML) is a clonal disorder characterized by the presence of immature blasts and arrested differentiation.^1^ AML is primarily a bone marrow (BM) disease, but AML cells can emerge in extramedullary (EM) sites, and some patients with AML have isolated EM disease without BM involvement.^2^ These EM manifestations highlight a highly adaptable leukemic process, shaped by signals from the surrounding microenvironment, and this process can be revealed through spatial analyses of both medullary and EM tissues. Uncovering the spatial dynamics of AML cells is essential for identifying therapeutic targets and understanding resistance mechanisms. Spatial proteomics analysis has provided mechanistic insights into the immune evasion of AML cells and has revealed subcellular compartments in AML cells.^3^^,^^4^ However, spatial proteomics can be constrained by a predetermined, targeted approach, which limits its breadth to a fixed set of proteins, thus making it more useful for validation than for novel discovery. In contrast, spatial transcriptomics (ST), which can capture a vast array of genes without bias, facilitates the discovery of biological pathways, molecular profiles, and cellular interactions. Although, high-throughput ST is widely applied in solid cancers,^5^^,^^6^^,^^7^ its use in BM-based diseases remains relatively limited,^8^^,^^9^^,^^10^^,^^11^ likely because the rigorous decalcification required for the processing and sectioning of BM specimens may compromise RNA integrity, and because many researchers perceive BM diseases as “liquid” cancers that lack a defined tissue architecture.^12^ Leveraging ST to dissect AML can yield valuable insights into the tissue composition and cell-cell interactions within the osseous and non-osseous niches that sustain AML growth.

In the present study, we employed Visium array-based ST in paired diagnostic BM and EM samples from patients with AML and GeoMx digital spatial profiling on an AML tissue microarray to optimize spatial analysis in AML and study cell-cell interactions. By spatially deconvolving the leukemic components using reference single-cell RNA sequencing (scRNA-seq) data from BM biopsies from AML and healthy donors, we created an ST map of medullary and EM leukemia. We integrated the ST annotations with a proteomic panel of functional and phenotypic markers for orthogonal validation. Our findings captured established AML biology patterns such as the CXCL12-CXCR4 interactions, validating our approach. In addition, we found inflammatory pathways intertwined with endosteal BM niches in the maturation states of AML populations. We also mapped the spatial distribution of the AML hierarchy, providing insights into its organization within the microenvironment. Our study demonstrates the feasibility of using integrated ST and proteomic approaches to analyze the BM of patients with AML and supports the broader applicability of these methods beyond AML.

## Results

### Comparative multi-omics analysis of acute myeloid leukemia patients’ bone marrow and extramedullary tissues

**Figure 1:**
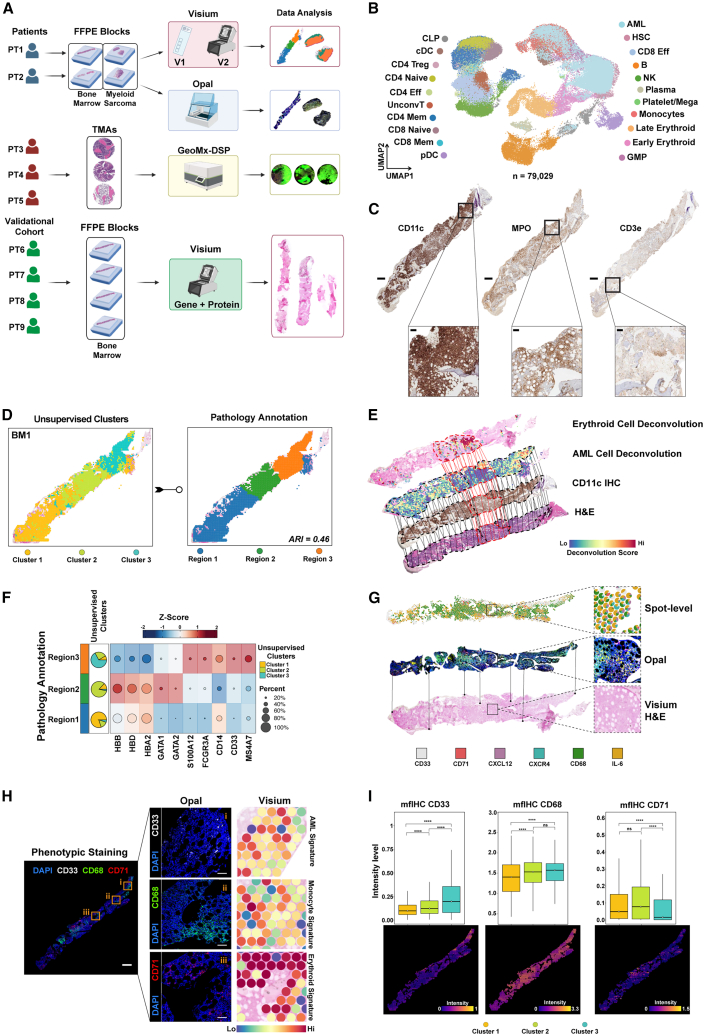
Comparative spatial multi-omics analysis of acute myeloid leukemia patients’ bone marrow and extramedullary tissues (A) Schematic representation of the study workflow. Paired bone marrow (BM) samples (BM1 and BM2) and extramedullary (EM) samples (EM1, from skin; and EM2, from lymph node) from 2 newly diagnosed patients with acute myeloid leukemia (AML) (PT1 and PT2) were fixed in formalin and embedded in paraffin (FFPE) and then sectioned for use in Visium assays (v1 and v2), and Opal multiplex fluorescent immunohistochemistry (mfIHC). The Visium spatial transcriptomics (ST) results were validated using GeoMx digital spatial profiling (DSP) with tissue microarrays (TMAs) of samples from 3 newly diagnosed patients with AML (PT3, PT4, and PT5). An additional 4 AML bone marrow samples that performed the Visium gene and protein expression assay are used as a validational cohort (PT6, PT7, PT8, and PT9). Image created with BioRender (https://biorender.com). (B) Uniform manifold approximation and projection (UMAP) plot showing our reference map consisted of 79,029 cells collected from 9 healthy BM donors and 7 patients with AML with diploid cytogenetics to match the patient cytogenetic profiles, and included both newly generated scRNA data and previous works. This map consisted of 21 cell types, including T cells (CD4^+^ and CD8^+^ naive, effector, and memory T cells, T regulatory [Treg] cells, and unconventional T cells), other immune cells (Natural killer [NK] cells, B cells and plasma cells), hematopoietic progenitors (Hematopoietic stem cells [HSCs], common lymphoid progenitors [CLPs], granulocyte-monocyte progenitors [GMPs]), myeloid cells (megakaryocytes/platelets, monocytes, early and late erythroid cells, conventional and plasmacytoid dendritic cells) and leukemic (AML) cell populations. (C) Immunohistochemical staining of CD11c, MPO, and CD3e on BM1 sections that were used for histopathological annotation. The scale bar for the main tissue panels represents 1 mm. The scale bar for the zoomed-in panels, corresponding to the boxed regions, represents 100 μm. (D) Unsupervised clustering and pathology annotation for the projected spatial map of BM1, revealing 3 distinct regions with an adjusted rand index (ARI) of 0.46. (E) Spatial deconvolution of BM1 tissue, showing erythroid and AML cell populations, with CD11c immunohistochemistry (IHC) overlaid on an image of hematoxylin and eosin (H&E)-staining. The dotted red lines represent regions enriched for the erythroid cell population; dotted black lines, regions enriched for the AML cell population; and solid lines, regions that overlapped with other tissue sections. (F) Heatmap of *Z* score normalized canonical markers in pathology annotations, with matching unsupervised cluster distributions represented as a pie chart. *HBB, HBD, HBA2, GATA1/2* are erythroid genes and *S100A12, FCGR3A, CD14, MS4A7,* and*, CD33* are monocyte/leukemic genes. (G) Representative overlay of Visium H&E staining with Opal mfIHC and the generated spot-level data for CD33, CD71, CXCL12, CXCR4, CD68, and IL-6. Boxes illustrate magnified regions showing concordance between transcript-level (Visium) and protein-level (Opal) signals at the spot level. (H) Phenotype staining on near-adjacent tissue sections for markers of leukemic (CD33), monocytic (CD68), and erythroid (CD71) populations. DAPI was used as a nuclear counterstain. The spatial distribution of these markers corroborates ST-based spot deconvolution. Scale bars: 1 mm (whole-slide panels) and 100 μm (selected region panels). (I) Box and spatial plots of mfIHC staining intensities for phenotypic markers across ST-defined clusters in BM1, highlighting the enrichment of leukemic and monocytic populations in cluster 3 and that of erythroid populations in cluster 2 at BM1. Scale bars: 1 mm (whole-slide panels) and 100 μm (selected region panels). ns, not significant. ∗∗∗∗*p* < 0.0001, Wilcoxon rank-sum test.

Our objective was to characterize spatial interactions in AML while addressing the challenges posed by the decalcification process and RNA quality inherent to the histopathological processing of BM samples. To establish a control, we hypothesized that pairing BM tissues with EM non-osseous AML from the same patient would serve as an optimal comparison. We identified diagnostic trephine BM and EM biopsy samples collected from 2 patients with AML to be used for Visium-based ST profiling and 4 AML patient BMs as validation cohort with the Visium gene and protein expression assay (Figure 1A). The patients’ clinical characteristics are summarized in Table S1. Briefly, one patient, and 83-year-old man, presented with a cutaneous myeloid sarcoma (sample EM1) and concurrent medullary leukemia (30% myeloblasts, sample BM1), and the other patient, a 42-year-old man had a mediastinal mass (sample EM2) without histopathologic evidence of medullary AML involvement (2% myeloblasts, sample BM2) (Figures S1A–S1C). BM1 harbored mutations in *NPM1, DNMT3A, IDH1, IDH2, FLT3, SF3B1, and KRAS*. The targeted mutation panel was not performed for BM2 or EM2. Both patients had diploid cytogenetics.

We tested 2 Visium-based assays: version 1 (v1), and version 2 (v2) (Methods) using all 4 patient samples (BM1, BM2, EM1, and EM2) concurrently. The pre-library RNA traces (DV_200_ values) were 39% or higher for all samples except EM2 (24%) (Figures S1D–S1F). For BM1 and BM2, the post-library DNA traces in v1 (24% and 29%) were markedly lower than those in v2 (89% and 91%, respectively) which precluded further sequencing of the v1 BM libraries, whereas for EM1 and EM2, the post-library DNA traces in v1 (87% and 83%, respectively) were similar to those in v2 (88% and 91%, respectively) (Figures S1E, S1G, and S1H). These findings suggest that Visium v2, which includes automated tissue transfer, performs more reliably than v1 with BM tissues, even those with low pre-library DV_200_ values (Methods). Also, v2 detected significantly more genes than v1 did (Figures S1I–S1M). However, spatial correlation between the Visium gene and protein expressions was weak, primarily due to low quality of the protein assay. (Figure S1N). To assess whether the structural integrity of bone regions was maintained during tissue processing, we overlaid the image of the BM section on the Visium slide with the image of the H&E-stained section. Indeed, spots in bone trabeculae areas, which usually have low cell abundance and a tendency to come off during tissue processing, were still maintained (Figure S1O). Overall, the v2 data demonstrated superior quality compared to v1, largely due to the automated tissue transfer approach, which preserved tissue integrity and thereby facilitated successful downstream analysis.

A known limitation of Visium is that multiple cells can occupy the same spot. Therefore, we utilized our in-house generated scRNA-seq BM reference data^13^^,^^14^^,^^15^^,^^16^ (Figure 1B) to deconvolve individual spots (spatial regions) using a probabilistic label transfer workflow. We next conducted shared nearest neighbor (SNN) modularity optimization-based unsupervised clustering.^17^ The resulting cluster labels were then compared to the tissue annotations established by 2 independent hematopathologists by overlaying the Visium slides with the H&E− and clinical IHC-stained slides (Figures 1C, 1D, S2A, and S2B). In BM1, the SNN-based clustering identified 3 distinct clusters. Regions were defined as “mixed” (Region 1), “erythroid-enriched” (Region 2), and “monocytic/leukemia-enriched” (Region 3) (Figure 1E). The results aligned with differentially expressed genes in these regions and canonical markers of erythroid cells (*HBB, HBA2, GATA1,* and *GATA2*), monocytes (*S100A12, FCGR3A, CD14,* and *MS4A7*), and myeloid cells (*CD33*) (Figure 1F, Table S2). To further confirm the deconvolution-based spot annotation, we performed mfIHC on near-adjacent tissue sections, enabling spatial proteomic analysis at the single-cell resolution (Figure S2D). To overcome the BM tissues’ high autofluorescence due to bone components and fixation, we applied light-based quenching^18^ and manually excluded these areas in downstream analyses (Figure S2E). The mfIHC data were then aligned with the corresponding Visium samples for spot level integration (Figure 1G). Using the phenotypic markers – CD68 (for monocytic populations), CD71 (erythroid cells), and CD33 (leukemic populations) – we validated our annotation approach. There was a consistent positive correlation between deconvolved cell types and protein intensities compared to single gene-protein correlations in EM (Figure S2F). The cluster-based distribution of protein expression mirrored the ST data; in BM1, cluster 3 was enriched for leukemic and monocytic populations, and cluster 2 was enriched for erythroid cells (Figures 1H and 1I). In BM1, the most abundant cell type was AML cells, followed by monocytes, whereas in BM2, late and early erythroid cells, along with GMPs, were most common (Figure S2G). Compared with BM2 (no morphologic leukemia detected), BM1 (∼30% leukemic blasts) had a slightly higher prevalence of effector and memory T cell populations but a lower abundance of erythroid-lineage populations.

**Figure 2:**
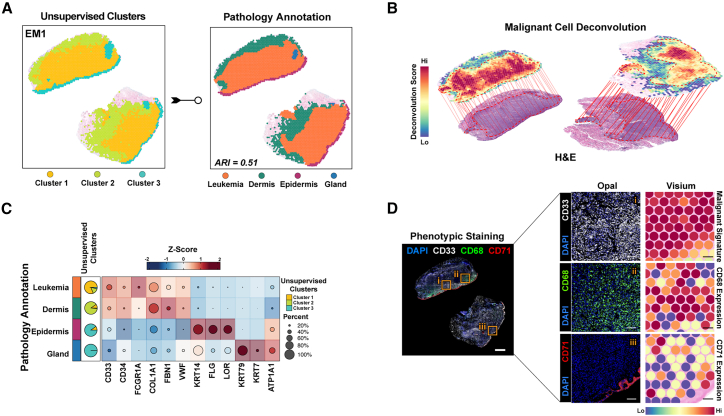
Spatial multi-omics profiling identifies leukemic infiltration and tissue composition in extramedullary acute myeloid leukemia samples (A) Unsupervised clustering of the extramedullary sample EM1 into 3 spatial clusters (left) compared against the pathology-based annotation (right; indicating a composition of leukemia, dermis, epidermis, and gland). The adjusted rand index (ARI; 0.51) reflects moderate agreement between the clusters and pathology annotations. (B) Spatial deconvolution scores obtained using the SpaCET algorithm show EM1’s malignant cell distribution overlaid on the hematoxylin and eosin (H&E) image. (C) Heatmap of canonical marker expression in EM1 regions, validating transcriptional segregation and matching pathologist-defined regions. Markers of leukemic populations and dermis regions show shared expression profiles. Unsupervised cluster overlap is represented as pie charts, with pathology annotation. (D) Phenotypic staining (Opal multiplex immunofluorescent) on near-adjacent sections validating the spatial distribution of CD33 (malignant cells), CD68, and CD71, which is consistent with the Visium malignant signature (spot-level) and CD68 and CD71 expression patterns. Scale bars: 1 mm (whole-slide panels) and 100 μm (selected region panels).

EM tissues included epidermis, dermis, germinal centers, and glands as expected due to the cutaneous nature of the EM disease. Unsupervised clustering segmented EM1 into 3 distinct clusters, which mostly overlapped with histopathological annotations, with the exception of glandular tissue glandular tissue annotations (*adjusted rand index = 0.51*) (Figure 2A). We then applied the spatial cellular estimator for tumors (*SpaCET*) algorithm,^19^ which is used in the spatial analysis of non-osseous solid cancer spatial analysis, to identify the leukemic regions (Figure 2B, STAR Methods). Deconvolution results were confirmed by comparing them with the histopathologic annotations on digital images of H&E staining of the same slide. Macrophages were the predominant predicted cell type in EM1, while cancer-associated fibroblasts and endothelial cells exhibited the highest abundance in EM2 (Figure S2H). Tissue-specific markers and differentially expressed genes validated the transcriptional segregation, revealing dermis infiltration by leukemic cells’ infiltration of the dermis in EM1, and confirmed the consistency of the unsupervised clusters with the pathologist-defined regions (Figures 2C and S2I; Table S3), which was also validated by mfIHC (Figure 2D). These results support the use of deconvolution for spot-level annotation, enabling further downstream analysis.

### Spatial heterogeneity of cell populations in bone marrow and extramedullary tissues in patients with acute myeloid leukemia

**Figure 3:**
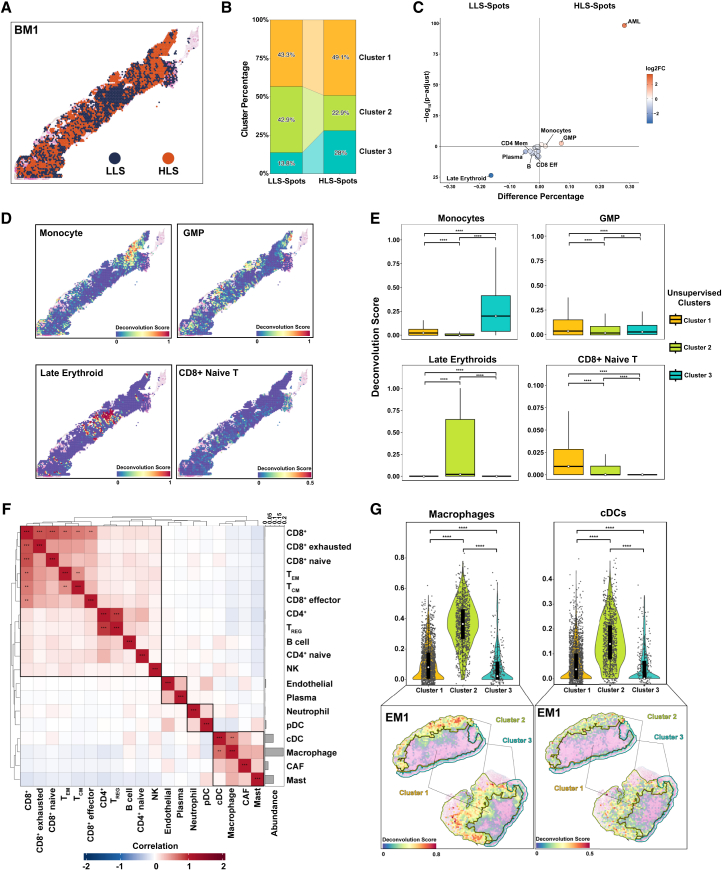
Spatial heterogeneity of acute myeloid leukemia populations in bone marrow and extramedullary tissues (A) Spatial map of the bone marrow sample BM1 showing spots with high leukemic scores (HLS; above the median acute myeloid leukemia [AML] deconvolution score; >0.15; orange) and low leukemic scores (LLS; below the median AML deconvolution score; ≤0.15; dark blue). (B) Stacked bar plot of the cluster-based distribution of HLS and LLS spots in BM1. (C) Volcano plot of the differential co-localization of cell populations within HLS and LLS spots of all BM samples. Deconvolution scores were compared using the Wilcoxon rank-sum test. (D) Spatial deconvolution of monocytes, and granulocyte-monocyte progenitors (GMPs) in HLS spots in BM1 (top) and spatial deconvolution of late erythroid cells and CD8^+^ naive T cells in LLS spots in BM1 (bottom). (E) Boxplots of deconvolution scores for monocytes, GMPs, late erythroid cells, and CD8^+^ naive T cells across the 3 unsupervised clusters in BM1. Median values are shown as white dots on black lines. ∗∗*p* < 0.01, ∗∗∗∗*p* < 0.0001, Wilcoxon rank-sum test. (F) Correlation heatmap of cell populations in the extramedullary sample EM1, highlighting significant co-localization between macrophages and classical dendritic cells (cDCs).T_EM_, effector memory T cells; T_CM_, central memory T cells; NK, natural killer cells; pDC, plasmacytoid dendritic cells; CAF, cancer-associated fibroblasts. ∗∗∗r > 0.7, ∗∗r > 0.5, Pearson absolute correlation. (G) Spatial mapping of macrophage and cDC spots in EM1, showing their co-localization in tumor-infiltrated dermis clusters (clusters 1 and 2). ∗*p* < 0.05, ∗∗*p* < 0.01, ∗∗∗*p* < 0.001, ∗∗∗∗*p* < 0.0001, Wilcoxon rank-sum test.

We categorized each spot as having a high leukemic score (HLS; above the median AML deconvolution score) or a low leukemic score (LLS; below the median AML deconvolution score) (Figures 3A and S3A; Table S4). In BM1, cluster-based analysis showed that HLS spots were about 14% more frequent than LLS spots in cluster 3, whereas LLS spots were 20% more frequent than HLS spots in cluster 2 (Figure 3B). Monocytes and GMPs were predominantly associated with HLS spots in the BM environment (Figures 3C, S3B, and S3C). In addition, neighborhood analysis further showed that these cell types were also enriched in the immediate surroundings of HLS spots (Figure S3D). Monocytes were primarily in cluster 3, whereas GMPs were enriched in all 3 spatial clusters. CD8^+^ naive T cells were found throughout all clusters but had a higher spatial concentration in cluster 1. In cluster 2, late erythroid cells were predominant, and this region showed the lowest proportion of HLS-spots (Figures 3D and 3E). In EM1, immune cell populations were co-localized. For instance, macrophages, which had the highest abundance in EM1, had strong co-localization with classical dendritic cells (*Pearson correlation coefficient [r] = 0.67*) and were associated with cancer-associated fibroblasts (*r* = 0.37) (Figure 3F), particularly in the tumor-infiltrated dermis region (Figure 3G).

### Inferred pathway analysis reveals inflammatory niches and region-specific signatures in bone marrow and extramedullary tissues from patients with acute myeloid leukemia

We next compared the regulatory programs, distinguishing HLS spots from LLS spots (Figures S3E and S3F). Several genes involved in immune regulation, inflammasome activity, and tumor progression, such as CD70, TMEM176B, TP53INP2, and TNFSF13B,^20^^,^^21^^,^^22^^,^^23^^,^^24^ were significantly enriched in the HLS spots in BM1, and these same genes were also highly expressed in the EM leukemic regions of the same patient (Figure S3G). Differential expression analysis between BM HLS spots and EM malignant regions revealed enrichment of erythroid-lineage differentiation genes characteristic of low-cycling progenitors (e.g., HEMGN, KLF1, and ALAS2), whereas EM spots preferentially expressed extracellular matrix and EMT-associated genes (e.g., CIDEA, PLIN5, and CLMP) (Figure S3H). Pathway profiling further demonstrated that the paired BM and EM samples from each patient shared common molecular signatures (Figure S4A). For instance, in PT1, pathways related to inflammation (e.g., IFNα, IFNγ, inflammatory response, and TGF-β pathways) and energy metabolism (e.g., oxidative phosphorylation and glycolysis pathways) were upregulated in both BM1 and EM1, whereas these were relatively downregulated in both BM2 and EM2. We also observed prominent epithelial-mesenchymal transition (EMT)-like programs linked to neoplastic migration and *trans*-differentiation in the EM1 and EM2. Notably, the profiles of cluster 3 of BM1, particularly those involving inflammation-related pathways, closely matched those of the leukemic cluster of EM1 (Figure S4B).

**Figure 4:**
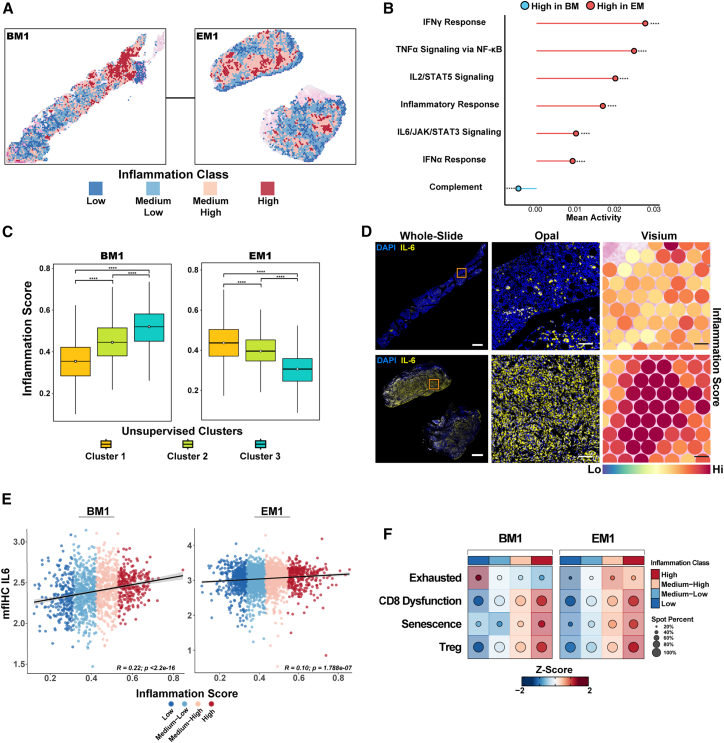
Inflammatory microenvironment analysis reveals region-specific signatures in bone marrow and extramedullary tissues from patients with acute myeloid leukemia (A) Distribution of spatial inflammation classes in BM1 and EM1, based on composite inflammation scores from inflammation-related hallmark pathways (Inflammatory response, IL6/JAK/STAT3 signaling, TNF-α/NF-κB signaling, IFN-γ response, IFN-α response, Complement, IL2/STAT5 signaling). Classes were defined using Jenks' natural breaks optimization. (B) Mean activity comparison of individual inflammatory related pathways in spots with high-inflammatory activity revealed the highest activity of IFN-γ response in EM tissue. Complement pathway activity is higher in BM1 when compared with the EM1 inflammatory niche. ∗*p* < 0.05, ∗∗*p* < 0.01, ∗∗∗*p* < 0.001, ∗∗∗∗*p* < 0.0001, Wilcoxon rank-sum test. (C) Boxplots of inflammation scores across the 3 clusters in BM1 (left) and EM1 (right). Each cluster displays significantly different levels of inflammatory activity; leukemia-enriched cluster 3 in BM1 and cluster 1 in EM1 have higher inflammation scores. ∗*p* < 0.05, ∗∗*p* < 0.01, ∗∗∗*p* < 0.001, ∗∗∗∗*p* < 0.0001, Wilcoxon rank-sum test. (D) IL-6 staining (Opal multiplex fluorescent immunohistochemistry [mfIHC]) in whole-slide images (left) of BM1 (top) and EM1 (bottom) and corresponding magnified regions (center), aligned with Visium spot-level composite inflammation score (right). Scale bars: 1 mm (whole-slide panels) and 100 μm (selected region panels). (E) Scatterplots showing the correlation of IL-6 protein staining intensity (mfIHC IL-6) with the composite inflammation score in BM1 (left) and EM1 (right). IL-6 levels are higher in high-inflammation regions in both BM1 and EM1. (F) Dot plot showing the localization of T cell subtypes (exhausted, CD8^+^ dysfunction, senescence, regulatory T cells [Treg]) based on inflammation class in BM1 and EM1.

Dysregulated inflammatory pathways in the BM microenvironment contribute to leukemogenesis and leukemic blast maintenance in AML.^25^^,^^26^ We thus defined a composite inflammation score using inflammation-related hallmark pathways and then clustered the spatial data using Jenks natural breaks optimization (STAR Methods, Figures 4A and S4C). When comparing only high-inflammatory regions, the EM site revealed greater inflammation (Figure S4D). Among the pathways constituting the composite inflammation score, IFN-γ signaling was specifically elevated in EM, whereas only the complement pathway showed higher activity in BM (Figure 4B). As expected, cluster 3 in BM1 and cluster 1 in EM1 exhibited the highest inflammation scores, which is consistent with the leukemic enrichment in these clusters (Figure 4C). To further explore the spatial inflammatory pathways, we performed mfIHC for IL-6, a key inflammatory marker, and found similar spatial patterns of IL-6 expression in adjacent regions (Figure 4D). Furthermore, for both BM1 and EM1, the IL-6 staining intensities were positively correlated with the composite inflammation score (Figures 4E and S4E).

Given the association between high inflammation and AML enrichment, we next sought to determine whether this highly inflammatory niche influences T cell states across bone marrow and extramedullary sites. Although T cells were relatively scarce in our spatial profile, deconvolution analysis revealed that T cells with exhausted phenotypes were generally enriched in high-inflammatory regions. However, in BM1 and BM6, exhausted phenotypes appeared more prominent in lower inflammatory regions. CD8^+^ dysfunction and T regulatory cell signatures were consistently associated with highly inflamed niches across all samples (Figures 4F, S4F, and S4G).^14^^,^^16^^,^^27^ Taken together, these observations suggest that BM and EM sites in the same patient can have similar inflammatory programs, which are associated with T cell exhaustion within highly inflammatory niches.

### Spatial cell communication analysis highlights the CXCL12-CXCR4 axis in the inflammatory niche

**Figure 5:**
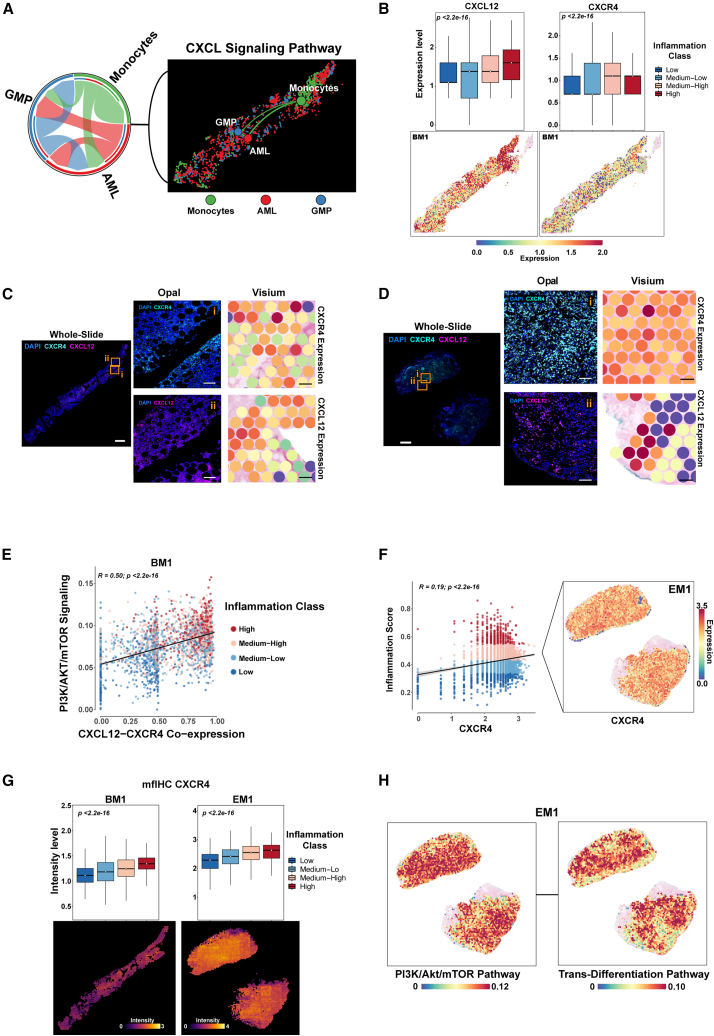
Chemokine signaling through the CXCL12-CXCR4 axis is linked to inflammatory niches and trans-differentiation in acute myeloid leukemia (A) Spatial and chord diagrams of the strength of interactions among acute myeloid leukemia (AML) cells, granulocyte-monocyte progenitors (GMP), and monocytes through the CXCL12-CXCR4 axis, as predicted by CellChat. (B) Boxplots of the expression levels of CXCL12 and CXCR4 in BM1, stratified by inflammation class (top), and corresponding spot-level expression maps (bottom) for the bone marrow sample BM1. Red spots indicate higher expression levels. (C and D) Whole-slide images of Opal multiplex fluorescent immunohistochemistry (mfIHC; left) for CXCR4 (turquoise) and CXCL12 (magenta) overlaid with DAPI (blue), alongside magnified Opal regions and Visium-based gene expression maps (right) in BM1 (C) and the extramedullary sample EM1 (D). Scale bars: 1 mm (whole-slide panels) and 100 μm (selected region panels). (E) Scatterplot shows the positive correlation of the PI3K/Akt/mTOR pathway score with the combined CXCL12-CXCR4 co-expression score (R = 0.50, *p* < 2.2e-16). Colors denote inflammation class. (F) Relationship between CXCR4 expression and inflammation score in EM1 (R = 0.19, *p* < 2.2e-16). Spatial maps show the distribution of CXCR4 expression. (G) Boxplots comparing CXCR4 protein signal intensity (mfIHC) across inflammation classes in BM1 (left) and EM1 (right). Spot-level images illustrate higher CXCR4 signal intensities in high-inflammation areas. (H) Sections 1 and 2 represent adjacent serial sections of the same EM1 biopsy embedded on a single Visium capture area. Visium ST visualization of PI3K/Akt/mTOR pathway (left) and *trans*-differentiation pathway (right) activity in these EM1 sections, revealing elevated pathway scores in high-inflammation and leukemic regions.

After defining each cell type based on the deconvolution score, we performed spatial cell-cell communication analysis (Figure S5A).^28^ Among the most prominent signaling interactions, we identified that strong CXC chemokine family (CXCL) signaling was present across all subtypes (Figure S5B). For instance, the CXCL12-CXCR4 axis emerged as a key pathway among AML cells, GMPs, and monocytes (Figures 5A, S5C, and S5D). Examining the relationship of the CXCL12-CXCR4 pair within the inflammatory niche, we found that both the CXCL12 ligand and the CXCR4 receptor showed high expression levels in spots with higher inflammation scores (Figures 5B, S6A, and S6B). To validate our ST findings, we performed mfIHC for the CXCL12-CXCR4 axis (Figures 5C, 5D, S6C, and S6D). In EM1 samples, both CXCL12 and CXCR4 demonstrated spatial concordance between gene expression and protein localization. In BM, CXCL12 showed high concordance, while CXCR4 exhibited minimal spatial overlap. In these highly inflammatory regions, the PI3K/AKT/mTOR pathway, a downstream target of the CXCL12-CXCR4 axis, was strongly correlated with the co-expression of CXCL12-CXCR4 (Figures 5E and S6E–S6H).^29^ This pathway’s ability to directly induce *trans*-differentiation aligns with our observation of elevated epithelial-mesenchymal transition (EMT) programs in these areas (Figures S6I and S6J).

To examine whether this mechanism was linked to the EM sites, we assessed the spatial expression of CXCL12 and CXCR4 in EM1. CXCR4 was abundantly expressed throughout the tissue and positively correlated with the composite inflammation score (Figure 5F), and spatial proteomics demonstrated increased CXCR4 protein levels in the inflammatory regions of EM1 (Figure 5G). By contrast, CXCL12 levels were elevated in the inflammatory regions of BM1 but were more diffusely distributed in EM1 (Figures S6K and S6L). Notably, both PI3K/AKT/mTOR signaling and *trans*-differentiation programs were active in the highly inflammatory regions of EM1 (Figure 5H).

### Deconvolution of leukemia-enriched spots reveals the localization of acute myeloid leukemia cells in different differentiation states within inflammatory and endosteal niches

**Figure 6:**
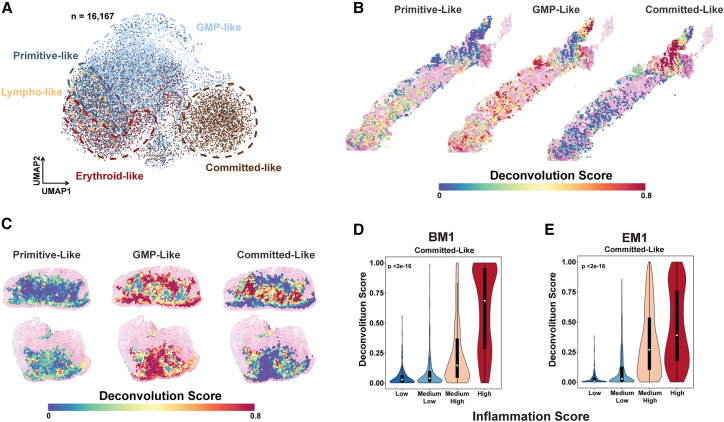
Hierarchical differentiation states of acute myeloid leukemia cells and their distribution across bone marrow and extramedullary tissues (A) Uniform manifold approximation and projection (UMAP) projection of 16,167 acute myeloid leukemia (AML) cells into different differentiation states: primitive-like, granulocyte-monocyte progenitor (GMP)-like, erythroid-like and lymphoid-like, and committed-like. (B) Spatial deconvolution maps of spots with high leukemic scores in the bone marrow sample BM1 showing primitive-like, GMP-like, and committed-like AML cells. (C) Spatial deconvolution maps of the extramedullary sample EM1 showing primitive-like, GMP-like, and committed-like AML cells. (D and E) Violin plots shows the distribution of committed-like AML cells in BM1 (D) and EM1 (E) across inflammation classes.

We next applied linear mixed model annotation^30^ to classify AML cells (*n* = 16,167 cells) based on their scRNA data into their differentiation states relative to the hierarchies in control BM samples from healthy donors (*n* = 20,778 cells).^31^ This classification defined AML cells as: primitive-like (a combination of HSC-like and common myeloid progenitor/lymphoid-primed multipotent progenitor-like; *n* = 5,039), GMP-like (*n* = 6,432), erythroid-like (*n* = 1,816), lymphoid-like (*n* = 55), and committed-like (a combination of monocyte-like, basophil-like, and dendritic cell-like; *n* = 2,825) (Figure 6A). We then applied this classification to HLS spots in BM1 (*n* = 1,271 spots) (Figure S7A) and leukemic region spots in EM1 (*n* = 1,726 spots) (Figures 6B and 6C) to obtain a spot-level spatial classification of AML hierarchies. Cluster-based analysis revealed that committed-like populations were located distally to the primitive-like populations (Figures S7B and S7C). Deconvolution scores indicated a lower abundance of primitive-like cells compared to committed-like cells in EM tissue (Figures S7D and S7E). We found that committed-like AML populations were concentrated in inflammatory niches (Figures 6D, 6E, and S7F).

**Figure 7:**
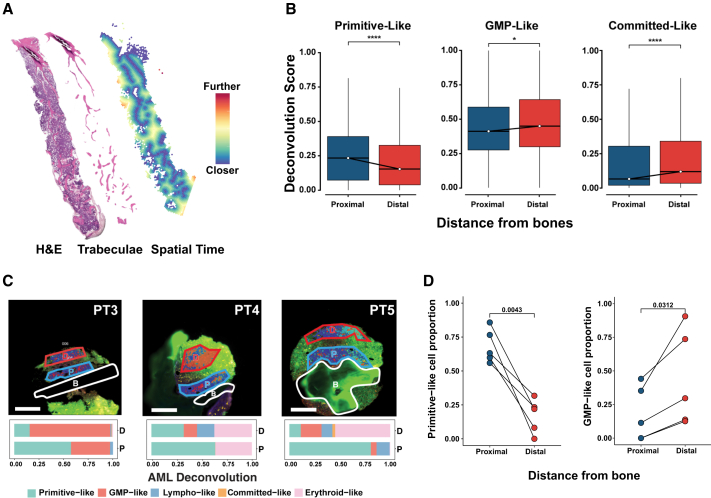
Bone proximity analysis reveals the spatial distribution of acute myeloid leukemia cells in different differentiation states (A) Representative spatial map of SpatialTime calculated distances from trabeculae overlaid with hematoxylin and eosin (H&E) image. (B) Boxplots show deconvolution scores of primitive-like, granulocyte-monocyte progenitor (GMP)-like, and committed-like acute myeloid leukemia (AML) cells relative to their distance from bone in Visium data. ∗*p* < 0.05, ∗∗∗∗*p* < 0.0001, Wilcoxon rank-sum test. (C) GeoMx analysis of AML deconvolution in bone marrow regions from 3 patients with AML (PT3, PT4, PT5). D, distal (dark red); P, proximal (dark blue); B, bone (white). Stacked bar plots represent cell type deconvolution within distal and proximal regions. Scale bars: 250 μm. (D) Line graphs show proportions of primitive-like and GMP-like cells relative to distance from bone.

We then applied the SpatialTime pipeline^32^ (Methods) to measure the spatial localization of HLS spots, based on the degree of AML differentiation, relative to the trabecular bone regions (Figure 7A; Figure S7G). We found that primitive-like cell populations were localized proximally to the bone, whereas GMP- and committed-like populations were localized distally (Figure 7B). To validate these findings, we performed a GeoMx DSP whole-transcriptome microdissection-based assay on 13 BM regions from 3 additional patients with AML. (Figures S7H and S7I). CD34 and CD68 protein markers were used to define the leukemic regions covering primitive and more differentiated cells (Figures S7J and S7K). Of note, we also attempted CD3 for T cell classification, but reliable segmentation mask could not be generated. Congruent with our Visium ST analysis, phenotypically primitive-like cells were detected proximal to the bone, whereas more differentiated cells were predominantly distal from the bone (Figures 7C and 7D). Taken together, these findings suggest that AML cells in different states of differentiation localize in distinct niches within the BM.

## Discussion

In this study, we demonstrated the feasibility of applying Visium ST to both medullary and EM AML tissues. By using the v2 assay, which has automated tissue transfer, we achieved better library quality, facilitating more robust downstream analyses. We also integrated our ST data with mfIHC data, illustrating the value of combining transcriptomic and proteomic information, which represents a key application of this integration for the Visium ST data with mfIHC in the analysis of AML BM. While next-generation imaging-based platforms such as Xenium and MERSCOPE offer higher resolution, they rely on targeted panels that are best suited for validation.^33^ Our approach provides a broad, discovery-oriented snapshot of AML’s spatial landscape. In addition, we complemented our Visium approach with GeoMx-based DSP to orthogonally validate its features, underscoring Visium’s potential applicability to other BM malignancies.

Inflammation is a well-established hallmark of cancer,^34^ and recent work indicates that inflammatory states shape the immune microenvironment, are correlated with AML differentiation, and impact disease progression and chemoresistance.^8^^,^^13^^,^^26^^,^^35^^,^^36^ In our spatial analyses, we classified Visium spots by their inflammatory signatures and uncovered distinct “niches” in both BM and EM tissues. Notably, regions with higher inflammation hosted AML cells spanning multiple differentiation states and showed a pronounced association between committed-like AML cells and inflammatory signals.

We observed that highly inflammatory niches often harbored AML cells and monocytes, highlighting the CXCL12-CXCR4 axis as a central signaling pathway. CXCL12 binds to CXCR4 and governs AML cell homing, migration, and therapy evasion in the BM.^37^^,^^38^^,^^39^^,^^40^ Our EM samples had widespread CXCR4 expression, suggesting that this pathway may also facilitate leukemic infiltration beyond the BM.

Downstream of the CXCL12-CXCR4 axis is the PI3K/AKT/mTOR pathway, which is well known to promote EMT-like processes in solid tumors.^29^^,^^41^^,^^42^^,^^43^ In our study, EMT-like or *trans*-differentiation signatures were correlated with CXCL12-CXCR4 signaling in inflammatory niches, suggesting a possible mechanism by which AML cells disseminate along the medullary-EM axis, consistent with recent findings implicating EMT pathways in EM AML progression.^44^ These findings warrant further investigation, particularly in patients with concomitant EM disease. CXCR4 inhibitors have been evaluated previously in AML. Our spatial results suggest revisiting CXCL12/CXCR4 targeting with biomarker guidance and considering combinations with PI3K/AKT/mTOR inhibitors in inflamed niches.^41^

The BM-resident leukemic population closely resembled its EM-resident counterpart at the transcriptomic level, particularly within highly inflammatory niches. Genes such as CD70, RAB3D, and TP53INP2, along with novel markers such as TNFSF13B and TMEM176B, were upregulated in monocyte-like AML clusters in both BM and EM samples. This finding suggests a conserved program across these distinct microenvironments and highlights candidate targets for further investigation.

The interplay between hematopoiesis and the endosteal niche maintains the quiescence and self-renewal of HSCs and the supportive capacity of the BM microenvironment.^45^^,^^46^ Previous studies established that HSCs and multipotent progenitors concentrate near the bone surface, whereas committed progenitors and differentiated cells occupy more distal regions.^47^ Building on these findings, we used a multimodal ST approach to map AML differentiation states in the BM. Our analyses indicate that GMP-like and committed-like AML populations cluster farther from endosteal surfaces, whereas primitive-like cells localize nearer to the bone, implying that osteoblastic regions may help sustain AML stemness.

Our study represents one of the first in-depth applications of ST to paired medullary and EM AML samples. Recent publications have highlighted the technical challenges and quality control aspects of performing ST on human BM but have rarely addressed the extended biological insights that can emerge from such analyses.^9^ By contrast, we integrated Visium ST with mfIHC and DSP to not only tackle these technical hurdles but also to delve more deeply into AML’s spatial biology.

Integrating spatial multi-omics with emerging therapeutic strategies could provide a roadmap for precision interventions in AML. Spatial mapping of resistance-associated phenotypes, including immune evasion and inflammatory programs, may also guide the deployment of targeted immunotherapies in microenvironmentally and anatomically defined contexts. These applications underscore the translational relevance of spatial omics for tailoring therapy to microenvironmental heterogeneity.

In summary, our approach yielded key insights into the roles of inflammatory niches and the CXCL12-CXCR4-PI3K/AKT/mTOR axis in AML progression, including the possibility that the endosteal niche supports more primitive AML populations. Studies in larger, more diverse cohorts are necessary to validate and extend these observations. Nevertheless, our work underscores the potential of integrating ST with orthogonal assays to elucidate AML biology, potentially informing novel therapeutic avenues.

### Limitations of the study

Our study had certain limitations. Its small sample size reflects both the rarity of paired pre-treatment medullary and EM biopsy samples from patients with AML and the high cost of spatial assays. Moreover, our scRNA-seq reference data excluded neutrophils and mesenchymal stromal cells owing to challenges in isolating these cell types; in addition, the 55 μm spot size of the Visium assay can obscure finer details in highly heterogeneous tissues such as BM. While we employed a label transfer approach using scRNA-seq data to infer probabilistic cell-type scores across Visium spots, the lack of single-cell resolution limits the accurate detection of some populations, including the stromal cells that were missing in our reference map. To mitigate these issues, we employed a tailored median absolute deviation-based filtering method, broad clustering to define intra-sample niches, validation of cell predictions via H&E staining and IHC by 2 independent hematopathologists, and the complementary use of mfIHC and DSP for greater resolution; however, it is important to note that the mfIHC and Visium datasets were derived from near-adjacent sections separated by two sequential 5 μm cuts, which may introduce spatial mismatches and contribute to imperfect correlation between transcriptomic and protein signals. In addition, in the four BM samples analyzed, Visium’s dual gene and protein expression assay, we observed limited gene-protein spatial correlation, precluding their use for robust proteomics validation. Importantly, tissue procurement vary across institutions and patients (from time to collection to fixation, decalcification methods etc), introducing technical heterogeneity that may affect RNA quality, spatial resolution, signal intensity, which is challenging to fully standardizse in studies using patient-derived samples.

## Resource availability

### Lead contact

Further information and requests for resources should be directed to the lead contact, Hussein A. Abbas (habbas@mdanderson.org).

### Materials availability

This study did not generate new or unique reagents.

### Data and code availability

• Data: Visium spatial transcriptomics data is publicly available at GEO: GSE279576.
• Code: All analysis scripts in this article are available at https://github.com/abbaslab/2025_Spatial_Profiling_in_Medullary_Extramedullary_Leukemia
• Additional Information: Any additional information required to reanalyze the data reported in this article is available from the lead contact upon request.

## Acknowledgments

H.A.A. is supported by the Physician Scientist Award and an 10.13039/100014535Individual Investigator Award (RP240287) from the 10.13039/100004917Cancer Prevention and Research Institute of Texas (CPRIT). This work was also by MD Anderson’s Physician-Scientist Training Program, by philanthropic funding from Energy Transfer and Diego-Osio Llerenas Fund. We would like to thank Sarah Bronson and Joe Munch in MD Anderson’s Research Medical Library for editing the article; Thomas Huynh and Arizona Nguyen in MD Anderson’s Department of Veterinary Services for helping prepare histologic samples; and Dr. Chong Wu in MD Anderson’s Department of Biostatistics for reading and providing comments on the article.

## Author contributions

H.A.A. conceived the study, supervised all aspects of the work, co-wrote, and reviewed the article. E.D. led the computational analyses and interpretation of Visium data. E.D., I.V., and H.A.A. wrote the article. I.V. and C.P.L. analyzed DSP data. C.D.P. performed mfIHC and analyzed mfIHC data. A.E.Q. and F.Z.J. performed pathology annotations. P.B., S.B., S.J., Z.W., A.L., and K.M.W. conducted the experiments and/or library preparation. D.A.A., P.H.G., P.K.R., P.S., and R.J.T. contributed conceptually to data analysis and design. All authors read and edited the article.

## Declaration of interests

H.A.A. received research support and Honorarium from Illumina and in-kind support from 10XGenomics; research support from GlaxoSmithKline, Ascentage, Taiho Pharmaceuticals, and Blueprint Medicines, Honorarium from Alamar Biotechnologies, and serves on advisory board of Cogent. P.S. received private investments from Adaptive Biotechnologies, BioNTech, JSL Health, Sporos, and Time Bioventures and served as a scientific advisory committee member for Achelois, Affini-T, Akoya Biosciences, Apricity, Asher Bio, BioAtla LLC, Candel Therapeutics, Catalio, C-Reveal Therapeutics, Dragonfly Therapeutics, Earli Inc, Enable Medicine, Glympse, Henlius/Hengenix, Hummingbird, ImaginAb, InterVenn Biosciences, LAVA Therapeutics, Lytix Biopharma, Marker Therapeutics, Matrisome, Oncolytics, Osteologic, PBM Capital, Phenomic AI, Polaris Pharma, Spotlight, Trained Therapeutix Discovery, Two Bear Capital, and Xilis, Inc. All other authors declare no relevant conflict of interest.

## STAR★Methods

### Key resources table

**Table:** 

REAGENT or RESOURCE	SOURCE	IDENTIFIER
**Antibodies**		

CD33 (clone PWS44) mouse mAb	CellMarque	Cat# 133M-14; RRID: AB_2861292
CD71 (clone H68.4) mouse mAb	Invitrogen	Cat# 13–6800; RRID: AB_2533029
IL-6 (clone 1.2-2B11-2G10) mouse mAb	Abcam	Cat# ab9324; RRID: AB_307175
CXCL12 (clone 79018) mouse mAb	R&D Systems	Cat# MAB350; RRID: AB_2088149
CXCR4 (clone D4Z7W) rabbit mAb	Cell Signaling Technology	Cat# 97680; RRID: AB_2800286
CD68 (clone PG-M1) mouse mAb	Agilent	Cat# M0876; RRID: AB_2074844
CD11c	MD Anderson Pathology Core	Clinical use; catalog not disclosed
MPO	MD Anderson Pathology Core	Clinical use; catalog not disclosed
CD3e	MD Anderson Pathology Core	Clinical use; catalog not disclosed

**Biological samples**		

Human bone marrow samples	The University of Texas MD Anderson Cancer Center	N/A
Human extramedullary leukemia samples	The University of Texas MD Anderson Cancer Center	N/A

**Critical commercial assays**		

Visium Spatial Gene Expression Slide & Reagent Kit	10x Genomics	PN-1000187
Visium Human Transcriptome Probe Panel v1	10x Genomics	PN-1000364
Visium CytAssist Slide & Casette Kit	10x Genomics	PN-1000518
Visium Human Transcriptome Probe Kit v2	10x Genomics	PN-1000466
Visium FFPE Reagent Kit v2	10x Genomics	PN-1000436
Visium Accessory Kit	10x Genomics	PN-1000194
Dual Index Kit TS Set A	10x Genomics	PN-1000251
Visium Human Immune Cell Profiling Panel	10x Genomics	PN-1000607
GeoMx Human Whole Transcriptome Atlas	NanoString	121401102
Opal 6-Plex Detection Kit	Akoya Biosciences	Cat# NEL871001KT

**Deposited data**		

Visium Spatial Transcriptomics Data	This paper	GEO: GSE279576
Opal Proteomics Data	This paper	https://github.com/abbaslab/2025_Spatial_Profiling_in_Medullary_Extramedullary_Leukemia

**Software and algorithms**		

SpaceRanger	10x Genomics	Version 2.0
GeomxTools	Bioconductor	Version 3.11.0
CIBERSORTx	Newman et al.^48^	https://cibersortx.stanford.edu/
Seurat	Hao et al.^5^^,^^49^	Version 5.0.3
Spatial Cellular Estimator for Tumors (SpaCET)	Ru et al.^19^	https://github.com/data2intelligence/SpaCET
AUCell	Aibar et al.^50^	https://github.com/aertslab/AUCell
CellChat	Jin et al.^28^	https://github.com/jinworks/CellChat
Symphony	Kang et al.^30^	https://github.com/immunogenomics/symphony
SCP version 0.5.1	Hao Zhang	https://github.com/zhanghao-njmu/SCP
ImageJ2	National Institutes of Health	https://github.com/imagej/imagej2
Visiopharm version 2024.07.1	Visiopharm A/S	https://visiopharm.com/
R version 4.3.1	The R Foundation for Statistical Computing	https://www.r-project.org/
Aperio ImageScope	Leica Biosystems	Version 12.4.6
ggplot2	R Core Team	Version 3.5.0

**Other**		

Aperio AT2 Digital Slide Scanner	Leica Biosystems	Model AT2
PhenoImager HT 2.0	Akoya Biosciences	Model HT2.0

### Experimental model and study participant details

#### Ethics statement

This study complied with the Declaration of Helsinki. Collection and use of human materials were approved by the Institutional Review Board of MD Anderson Cancer Center. (Institutional Review Board number: 2022-0576).

#### Human samples

The study included a total of 9 patients. Bone marrow (BM) and extramedullary (EM) core biopsy samples from 2 AML patients were used for the primary Visium comparison (BM1, EM1, BM2, EM2), 4 AML patient BMs were used for a validational cohort, and 3 AML patients were used for GeoMx DSP. The cohort consisted of 5 male and 4 female individuals. Due to the limited sample size within each group, the influence of sex/gender on the results could not be statistically determined. All patient clinical information is shown in Table S1.

### Method details

#### Sample preparation and clinical immunohistochemistry

BM and EM core biopsy samples were fixed in formalin and embedded in paraffin. (BM samples were decalcified with 10% formic acid before paraffin embedding.) Sections (4 μm) were cut for hematoxylin and eosin (H&E) staining and immunohistochemistry (IHC). For IHC, slides were deparaffinized, rehydrated, and subjected to heat-induced antigen retrieval. The slides were incubated with primary antibodies against CD11c, MPO, and CD3e for 1 h, incubated with horseradish peroxidase-conjugated secondary antibodies and 3,3′-diaminobenzidine, and counterstained with hematoxylin.

#### GeoMx digital spatial profiling

An 8x8 tissue microarray (TMA) was constructed from 12 FFPE bone marrow (BM) biopsies; nine cores were profiled using the NanoString GeoMx DSP platform. Two cores (AOIs 1–3) were excluded due to M6 AML. For selected cores, regions of interest (ROIs) were annotated both adjacent to and ≥200 μm distal from bone trabeculae and segmented into CD68^+^, CD34^+^, and non-myeloid AOIs using anti-CD68 (KP1), anti-CD34 (QBend/10), and SYTO13 staining. Anti-CD3 (PC3/188A) was included but failed quality thresholds and was not used. RNA expression (∼18,000 genes) was captured with UV-cleaved barcodes from the Whole Transcriptome Atlas. Data were processed with GeomxTools R package. Low-expressing probes were filtered, followed by Q3 normalization and log transformation.Myeloid AOIs were deconvolved using CIBERSORTx^4^ with a custom reference. Cell types were grouped into primitive-like, committed-like, and lymphoid-like states. Paired t-tests compared cell-type proportions between proximal and distal AOIs. Analyses were performed in R (v4.3.0).

#### Visium spatial transcriptomics

Formalin-fixed, paraffin-embedded BM and EM biopsy samples were processed using the Visium (10x Genomics). RNA quality was assessed as the percentage of fragments greater than 200 nucleotides (DV_200_). Tissue sections were processed via both the CytAssist platform (v2 assay; 11 × 11 mm capture areas) and directly placed on Visium slides (v1 assay; 6.5 × 6.5 mm capture areas). Four BM tissue sections were also profiled with the Visium Human Immune Cell Profiling Panel. Libraries were prepared according to standard protocols, SPRI-cleaned, quantified with Bioanalyzer and qPCR (KAPA kit), and sequenced on Illumina NovaSeq 6000. FASTQ files were generated and aligned to GRCh38 using SpaceRanger (v2.0). Tissue morphology was annotated by two expert pathologists based on H&E and IHC scans (Aperio, Akoya), and mapped to Visium spots via Loupe Browser.

#### Opal mfIHC

Near-adjacent FFPE sections from bone marrow and extramedullary tissues were processed for multiplexed fluorescent IHC using the Opal system (Akoya Biosciences). Slides were deparaffinized, underwent heat-induced epitope retrieval, and were sequentially stained with primary antibodies and Opal fluorophores, using horseradish peroxidase and iterative antibody stripping to enable multiplexing. DAPI was applied as a nuclear counterstain. Staining was automated on the NanoVIP 100 platform (Biogenex), and slides were imaged at 0.25 μm resolution using the PhenoImager HT 2.0. Spectral unmixing was performed at acquisition using custom fluorescence libraries. Unmixed images were aligned to Visium CytAssist reference images using 15–25 anatomical anchor points in Visiopharm TissueAlign. A single ROI per tissue was defined after segmenting and excluding bone, artifacts, and empty regions through a combination of deep-learning-based and manual refinement. Cell segmentation was performed based on DAPI signals using a U-net model. Marker intensities were smoothed, arcsinh-transformed, and averaged per cell, then aggregated to the Visium spot level based on spatial overlap. For spatial correlation analysis between transcript and protein signals, Lee’s L statistic was computed using Visium coordinates and a spatial weight matrix based on six nearest neighbors.

### Quantification and statistical analysis

All statistical analysis information can be found corresponded figure legends.

#### Filtering and data processing

Spatial spots were filtered by eliminating those not within ±3 median absolute deviations of mitochondrial gene content, total oligonucleotide counts, and detected gene numbers. Filtered spots underwent SCTransform normalization in Seurat, followed by principal component analysis and uniform manifold approximation and projection for dimensional reduction.

#### Spatial spot deconvolution

Reference scRNA seq data from BM samples from healthy donors (*n* = 9) and AML patients with diploid karyotype (*n* = 7) integrated. Visium BM samples from AML patients deconvolved with this reference by Seurat label transferring method. BM2 sample deconvolved with scRNA-seq reference contain only healthy compartments. Spatial spots were classified as having a high leukemic score (HLS; higher than the median AML deconvolution score) or low leukemic score (LLS; lower than the median AML deconvolution score). Due to structural differences EM samples were deconvolved with the SpaCET (Spatial Cellular Estimator for Tumors)^6^ Pan Cancer dictionary to identify cell types. AML cell states were further resolved using transfer learning.

#### Spatial analyses

Differential deconvolution analysis calculated with Wilcoxon rank-sum test between HLS and LLS spots for predicted cell type scores. Cell labels were assigned to spots based on prediction probabilities and used along with spatial coordinates to infer ligand-receptor interactions with the CellChat.^12^ To assess niche proximity, we applied SpatialTime pipeline.^48^ Trabecular bone regions were manually contoured, and the shortest distance from each Visium spot to the nearest bone surface was computed. Distances were scaled from 0 (adjacent) to 1 (furthest), and spots were classified as proximal or distal based on the median distance value. Spatial trends in cell states and pathway activity were analyzed relative to this spatial gradient.

#### Pathway analysis and inflammatory niche classification

Curated gene sets (hallmark) obtained from molecular signatures database and these gene sets were individually scored for each sample using the AUCell pipeline.^7^ Spatial coordinates and pathway scores for inflammatory pathways (IL6/JAK/STAT3 signaling, IFNγ Response, IFNα Response, TNFα/NF-κB signaling, complement, and IL2/STAT5 signaling) were extracted, normalized and a composite inflammatory score was calculated as the mean of these normalized scores. To stratify and define inflammatory niches, we applied the Jenks Natural Breaks classification method to divide the composite inflammation score into four categories (Low, Medium-Low, Medium-High, and High). This method optimally partitions continuous data by minimizing intra-class variance and maximizing inter-class variance, making it suitable for spatially skewed distributions without assuming cluster symmetry and size balance.

## Supplemental information

Document S1. Figures S1–S7 and Table S1

Table S2. BM1 unsupervised clusters differentially expressed gene list related to Figure 1F

Table S3. EM1 unsupervised clusters differentially expressed gene list related to Figure 2C

Table S4. BM1 Low Leukemic Score spots versus High Leukemic Score spots differentially expressed gene list related to Figure 3A

Table S5. OPAL multiplexed fluorescent immunohistochemistry antibodies related to Figure 1G-I, Figure 2D, Figure 4D–E, Figure 5C–D, G
