# Supplementary material for: Integrative spatial multi-omics reveal niche-specific inflammatory signaling and differentiation hierarchies in AML
Source: iScience. 2025 Nov 29;29(1):114289. doi: 10.1016/j.isci.2025.114289 (PMC12796006; doi:10.1016/j.isci.2025.114289)
Supplement: Document S1. Figures S1–S7 and Table S1 [file mmc1.pdf]

iScience, Volume *29*

Supplemental information

Integrative spatial multi-omics reveal niche-specific inflammatory signaling and differentiation hierarchies in AML

Enes Dasdemir, Ivo Veletic, Christopher P. Ly, Andres E. Quesada, Christopher D. Pacheco, Fatima Z. Jelloul, Pamella Borges, Sreyashi Basu, Sonali Jindal, Zhiqiang Wang, Alexander Lazar, Khalida M. Wani, Dinler A. Antunes, Patrick K. Reville, Preethi H. Gunaratne, Robert J. Tower, Padmanee Sharma, and Hussein A. Abbas

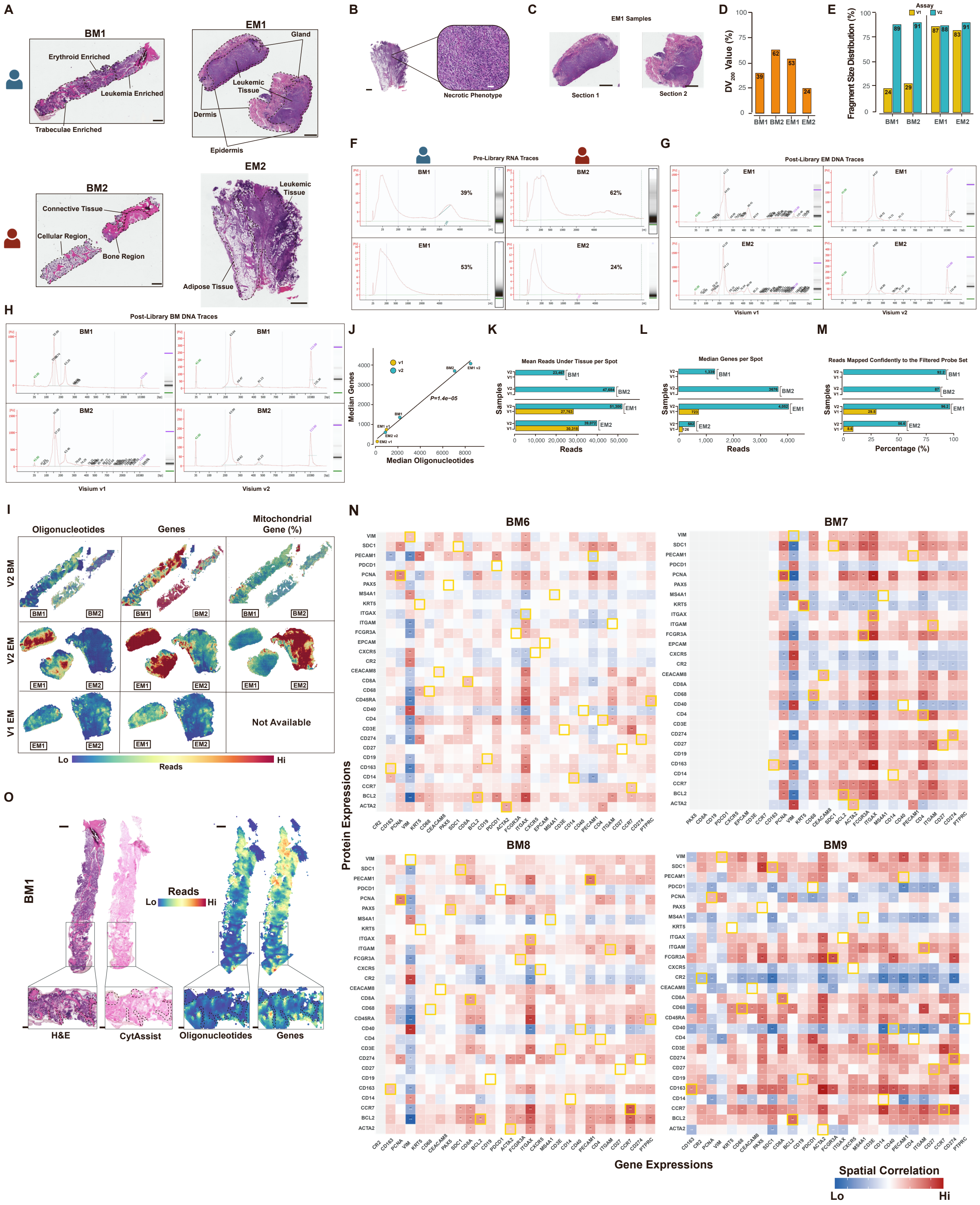

Figure S1: Evaluation of RNA and DNA integrity, and spatial transcriptomics quality metrics for bone marrow and extramedullary tissues from acute myeloid leukemia patients. (A) Histological sections of the bone marrow samples BM1 and BM2 and extramedullary samples EM1 and EM2 with hematoxylin and eosin (H&E) staining. Each section shows distinct tissue architecture and regions. The annotations are based on pathology annotations. Scale bars: 1 mm. (**B**) H&E-stained section showing the necrotic phenotype in EM2. While black scale bar at main tissue represents 1 mm, zoomed-in white scale bar represents 50 µm. **(C)** Two different samples of the extramedullary EM1 sample that were embedded in one paraffin block. Scale bars indicate 1 mm. **(D)** RNA fragmentation before Visium library preparation, measured as the percentage of fragments greater than 200 nucleotides (DV200), varied across the 4 analyzed samples (range, 24-62%; mean, 44.5%; standard deviation, 16.6%). EM2 showed the lowest pre-library

DV200 value (24%), whereas BM2 displayed the highest pre-library DV200 value (62%). **(E)** Fragment size distribution percentages calculated within the 200-1000 bp range for DNA libraries prepared using Visium assays (v1 and v2). BM1 and BM2 showed significant improvement with v2 compared to v1. EM1 and EM2 were within the acceptable range for both assays. (**F)** Pre-library RNA fragmentation traces (DV_{200} values) for BM1, BM2, EM1, and EM2. **(G)** Post-library DNA traces for EM1 and EM2 comparing v1 and v2. Similar DNA trace profiles were observed between v1 and v2 for both EM1 and EM2. (**H)** Post-library DNA traces for BM1 and BM2 comparing v1 and v2. Compared with v1, v2 displayed significantly higher post-library DNA traces. **(I)** Spatial mapping by Seurat of captured oligonucleotides, genes, and mitochondrial gene percentages in BM1, BM2, EM1, and EM2. Spatial mapping of oligonucleotide and gene counts obtained using the SpaceRanger pipeline revealed that compared to v1, v2 detected more oligonucleotides (mean nCount; 1292.76 for v1 vs 11072.37 for v2) and identified more genes (mean nFeature; 955.19 for v1 vs 4735.67 for v2) per sample. **(J)** There was a strong positive correlation between the median number of oligonucleotides detected and the median number of genes detected ($r = 0.996$, $p = 1.4 \times 10^{-5}$). (**K**) Comparison of mean reads under tissue per spot between v1 and v2 for BM1, BM2, EM1, and EM2. **(L)** Median genes per spot detected in v1 and v2 for BM1, BM2, EM1, and EM2. **(M)** Percentages of reads mapped confidently to the filtered probe set for v1 and v2 across the samples. **(N)** Lee's L spatial autocorrelation was performed to evaluate local correlation between transcriptomic and protein expression patterns across Visium capture spots. Yellow bordered cells highlight areas of correlation. Grey cells indicate no expression for corresponding gene and protein pair. **(O)** Histological overlay of an H&E-stained BM1 section with the CytAssist image and spatial transcriptomics data. The image shows the preserved structural integrity of bone regions, allowing detailed spatial analysis. The main tissue scale bar indicates 1 mm, while the zoomed-in panels, highlighting the boxed regions, denoting 400 μm.

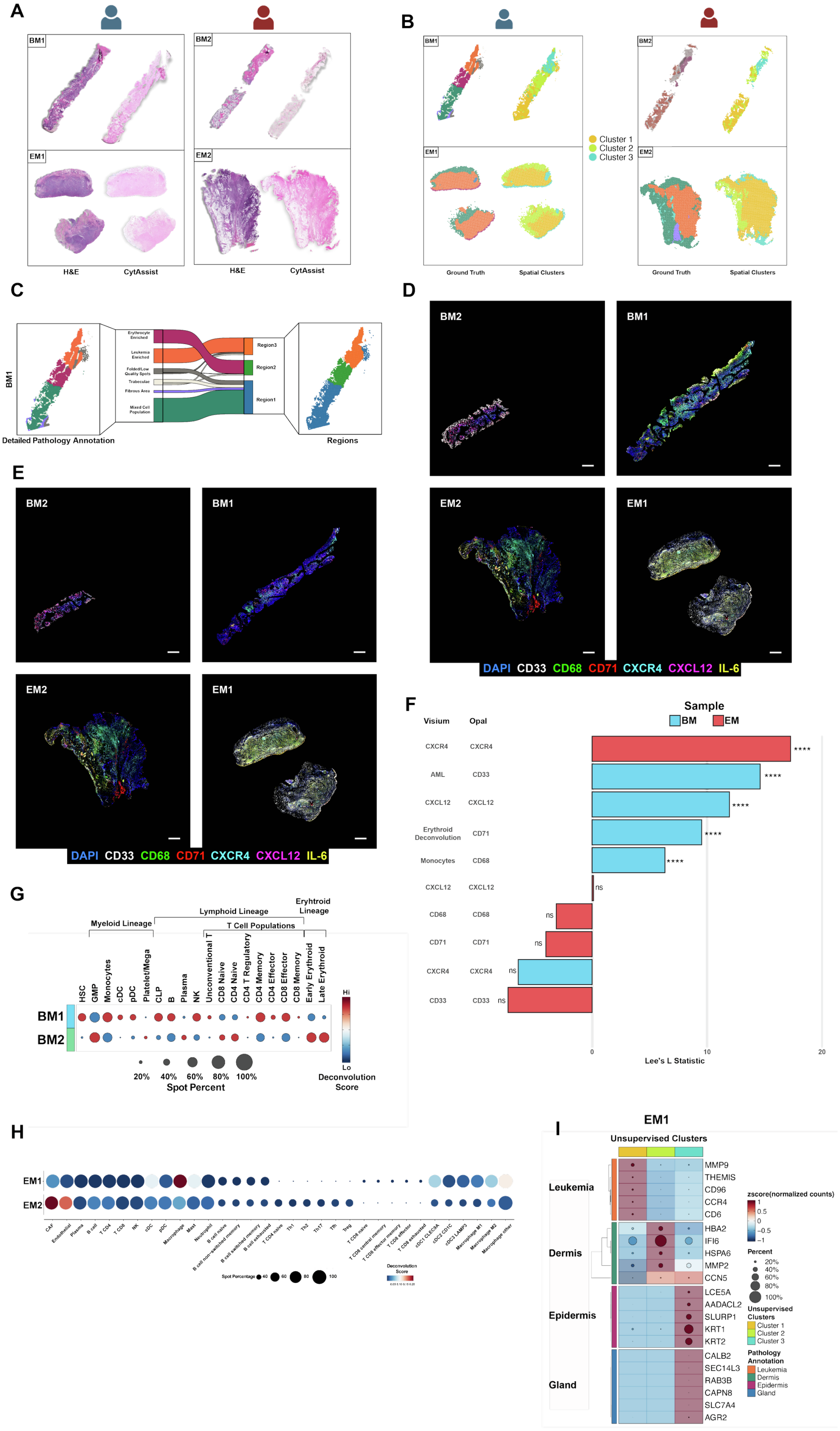
A
BM1
BM2
EM1
EM2
H&E
CytAssist
B
Cluster 1
Cluster 2
Cluster 3
Ground Truth
Spatial Clusters
C
BM1
Erythrocyte Enriched
Leukemia Enriched
Folded/Low Quality Spots
Trabeculae
Fibrous Area
Mixed Cell Population
Region3
Region2
Region1
Detailed Pathology Annotation
Regions
D
E
DAPI CD33 CD68 CD71 CXCR4 CXCL12 IL-6
F
Sample
BM
EM
Visium
Opal
CXCR4 CXCR4
AML CD33
CXCL12 CXCL12
Erythroid Deconvolution CD71
Monocytes CD68
CXCL12 CXCL12
CD68 CD68
CD71 CD71
CXCR4 CXCR4
CD33 CD33
****
ns
0
10
20
Lee's L Statistic
G
Myeloid Lineage
Lymphoid Lineage
T Cell Populations
Erythroid Lineage
HSC
GMP
Monocytes
cDC
pDC
Platelet/Mega
CLP
B
Plasma
NK
Unconventional T
CD8 Naive
CD4 Naive
CD4 T Regulatory
CD4 Memory
CD4 Effector
CD8 Effector
CD8 Memory
Early Erythroid
Late Erythroid
Hi
Lo
20%
40%
60%
80%
100%
Spot Percent
Deconvolution Score
H
EM1
EM2
Spot Percentage
Deconvolution Score
I
EM1
Unsupervised Clusters
Leukemia
Dermis
Epidermis
Gland
MMP9
THEMIS
CD96
CCR4
CD6
HBA2
IFI6
HSPA6
MMP2
CCN5
LCE5A
AADACL2
SLURP1
KRT1
KRT2
CALB2
SEC14L3
RAB3B
CAPN8
SLC7A4
AGR2
zscore(normalized counts)
Percent
20%
40%
60%
80%
100%
Unsupervised Clusters
Cluster 1
Cluster 2
Cluster 3
Pathology Annotation
Leukemia
Dermis
Epidermis
Gland

Figure S2: Integration of histopathological annotation, unsupervised clustering, and multiplex immunofluorescent staining for bone marrow and extramedullary tissues. (A) Hematoxylin and eosin (H&E) staining and CytAssist images of the bone marrow samples BM1 and BM2 and extramedullary samples EM1 and EM2. Scale bars: 1 mm. (**B**) Representation of ground truth and spatial clusters representations of the BM1, BM2, EM1, and EM2 samples. Unsupervised clustering segmented the tissues into distinct clusters. (**C**) Detailed pathology annotation of BM1. Areas are annotated as erythrocyte enriched areas, leukemia enriched areas, and other areas. The Sankey diagram illustrates the overlap between detailed pathology annotations and identified regions. (**D**) Raw Opal multiplex fluorescent immunohistochemistry (mfIHC) staining of BM1, BM2, EM1, and EM2. White indicates CD33; green, CD68; red, CD71; turquoise, CXCR4; magenta, CXCL12; and yellow, IL-6. Scale bars: 1 mm. (**E**) Filtered Opal mfIHC staining images for downstream analysis. Scale bars: 1 mm. (**F**) Spatial autocorrelation calculation between transcriptomic and protein-level signals across BM1 and EM1. Lee's L statistic was used to quantify the spatial concordance between Visium-derived deconvolution and gene expression and Opal-based mfIHC protein expression for selected marker pairs including chemokine-receptor (CXCL12-CXCR4), lineage (CD33 for AML, CD68 for monocytes, CD71 for erythroid cells). Asterisks indicate statistical significance of spatial correlation (**** $p < 0.0001$; ns: not significant). Bars represent Lee's L values for each marker pair in BM (blue) and EM (red) samples. Higher Lee's L values indicate stronger localized co-expression between transcriptomic and protein data across spatial coordinates. (**G**) Dot plot of cell type deconvolution in BM1 and BM2, highlighting differences among cell populations in each sample. HSC, hematopoietic stem cells; GMP, granulocyte-monocyte progenitors; cDC, classical dendritic cells; pDC, plasmacytoid dendritic cells; CLP, common lymphoid progenitors; NK, natural killer cells. (**H**) Dot plot showing the cell type deconvolution in EM1 and EM2, highlighting differences in cell populations between the two samples. CAF, cancer-associated fibroblasts; NK, natural killer cells; pDC, plasmacytoid dendritic cells; Th1, T helper 1 cells; Th2, T helper 2 cells; Th17, T helper 17 cells; Tfh, follicular helper T cells; Treg, regulatory T cells; cDC1 CLEC9A, conventional dendritic cell 1, CLEC9A+; cDC2 CD1C, conventional dendritic cell 2, CD1C+; cDC3 LAMP3, conventional dendritic cell 3, LAMP3+ (**I**) Heatmap of differentially expressed genes in EM1, annotated by unsupervised clusters.

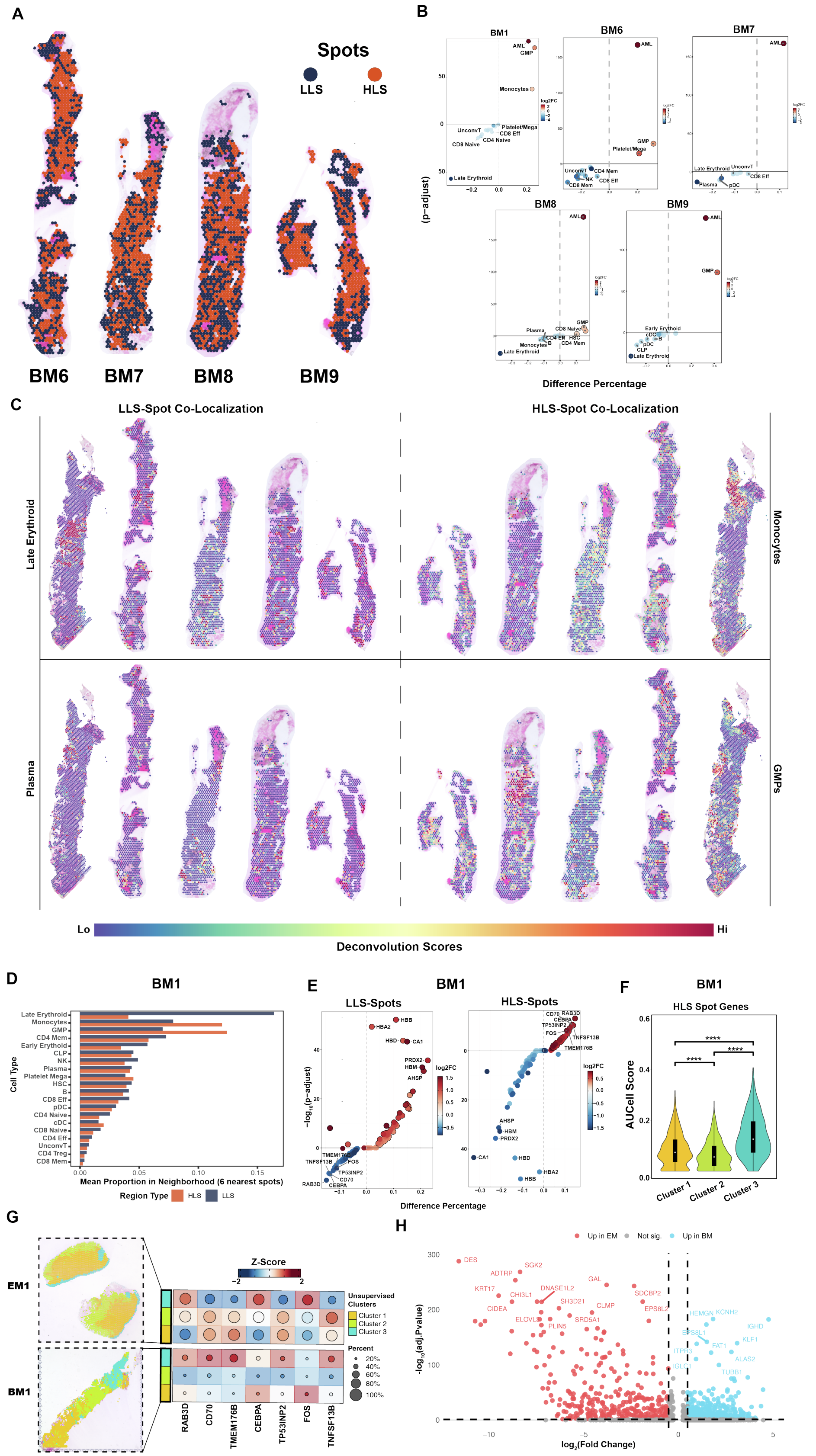

Figure S3: Comparison of leukemic compartments within medullary and extramedullary environments. (A) Spatial map of high-leukemic score (HLS) and low leukemic score (LLS) spots in validation cohort. Mean AML deconvolution results were used to dichotomized spots. (**B**) Differential deconvolution analysis between HLS and LLS spots revealed consistent co-localization between AML and GMPs in HLS spots. In contrast, late erythroid populations

significantly localized in LLS spots. (**C**) Spatial localization of the top two cell types that co-localized with HLS (monocytes and GMPs) and LLS (late erythroid and plasma cells) spots. (**D**) Neighborhood analysis of HLS and LLS spots showed a higher abundance of monocytes and GMPs surrounding HLS spots, and late erythroid cells surrounding LLS spots. Mean proportion in neighborhoods quantified the average composition of the six nearest spatial spots around each spot using probabilistic cell-type predictions. (**E**) Differential gene expression analysis of high leukemic score (HLS) spots and low leukemic score (LLS) spots in the bone marrow sample BM1. (**F**) AUCell score distribution of the top 15 HLS spot genes across 3 clusters, highlighting the concentration of HLS genes in cluster 3. ****$p < 0.0001$ (**G**) Heatmap of the results of differential gene expression analysis of high leukemic score (HLS) spots relative to low leukemic score (LLS) spots in the bone marrow sample BM1, highlighting genes associated with immune regulation and tumor progression. Genes highly expressed in the HLS spots of BM1, particularly in cluster 3, show an expression profile similar to that of the genes highly expressed in the leukemic-enriched cluster 1 of the extramedullary sample EM1. (**H**) Differential expression analysis between HLS spots in BM1 and pathology-assigned malignant spots of EM. The EM1 malignant spots showed significant enrichment of lipid and cholesterol metabolism-associated genes (e.g., CIDEA, PLIN5, FASN, ELOVL3/4), as well as extracellular matrix and epithelial-mesenchymal transition (EMT)-related genes (SDCBP2, TAGLN, COL1A1/2). In contrast, BM1 HLS spots were enriched for erythro-myeloid quiescence-associated markers (HEMGN, KLF1, ALAS2, KCNH2, IGHD).

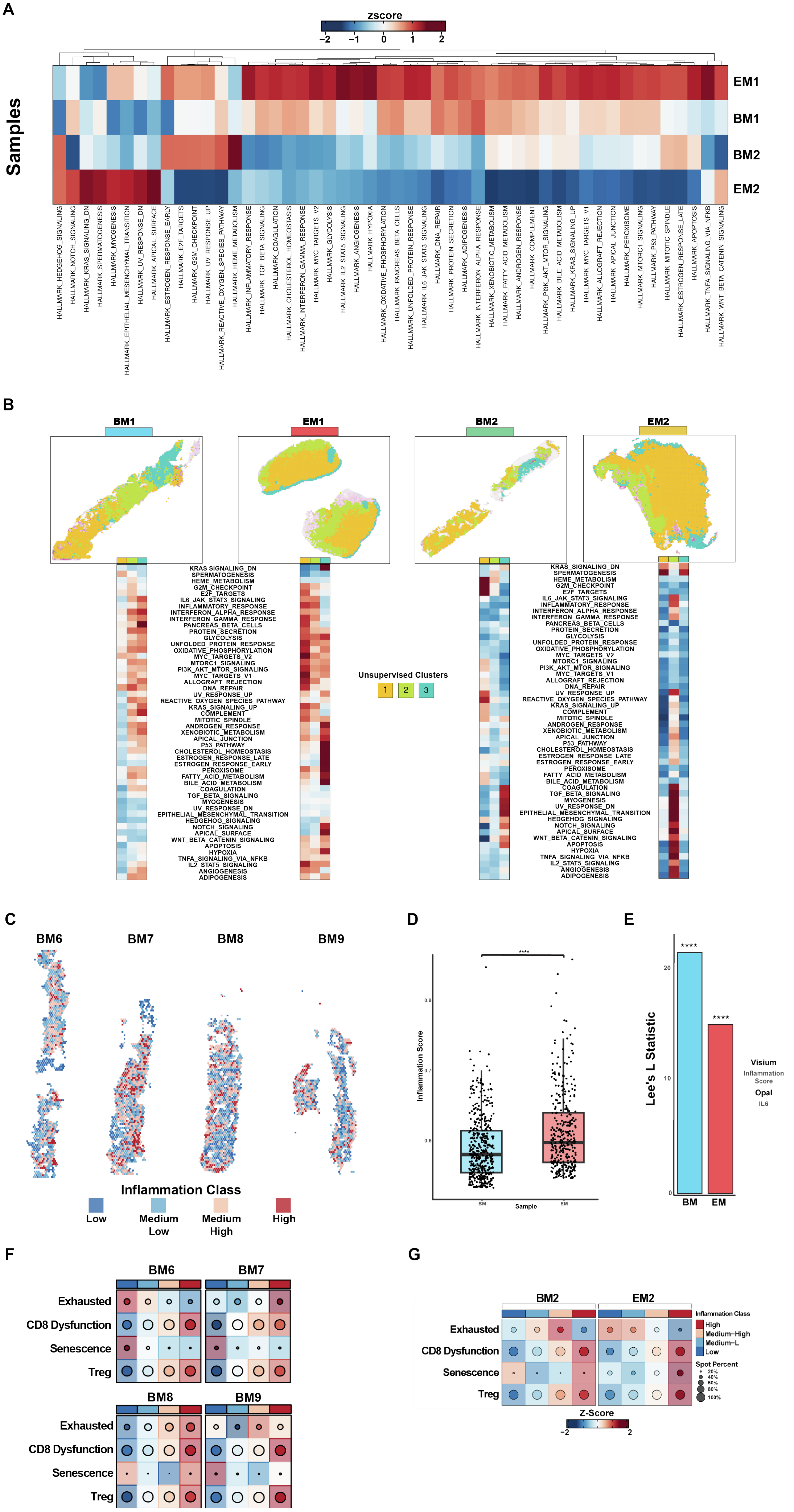
A
zscore
−2 −1 0 1 2
Samples
EM1
BM1
BM2
EM2
HALLMARK_HEDGEHOG_SIGNALING
HALLMARK_NOTCH_SIGNALING
HALLMARK_KRAS_SIGNALING_DN
HALLMARK_SPERMATOGENESIS
HALLMARK_MYOGENESIS
HALLMARK_EPITHELIAL_MESENCHYMAL_TRANSITION
HALLMARK_UV_RESPONSE_DN
HALLMARK_APICAL_SURFACE
HALLMARK_ESTROGEN_RESPONSE_EARLY
HALLMARK_E2F_TARGETS
HALLMARK_G2M_CHECKPOINT
HALLMARK_UV_RESPONSE_UP
HALLMARK_REACTIVE_OXYGEN_SPECIES_PATHWAY
HALLMARK_HEME_METABOLISM
HALLMARK_INFLAMMATORY_RESPONSE
HALLMARK_TGF_BETA_SIGNALING
HALLMARK_COAGULATION
HALLMARK_CHOLESTEROL_HOMEOSTASIS
HALLMARK_INTERFERON_GAMMA_RESPONSE
HALLMARK_MYC_TARGETS_V2
HALLMARK_GLYCOLYSIS
HALLMARK_IL2_STAT5_SIGNALING
HALLMARK_ANGIOGENESIS
HALLMARK_HYPOXIA
HALLMARK_OXIDATIVE_PHOSPHORYLATION
HALLMARK_PANCREAS_BETA_CELLS
HALLMARK_UNFOLDED_PROTEIN_RESPONSE
HALLMARK_IL6_JAK_STAT3_SIGNALING
HALLMARK_DNA_REPAIR
HALLMARK_PROTEIN_SECRETION
HALLMARK_ADIPOGENESIS
HALLMARK_INTERFERON_ALPHA_RESPONSE
HALLMARK_XENOBIOTIC_METABOLISM
HALLMARK_FATTY_ACID_METABOLISM
HALLMARK_ANDROGEN_RESPONSE
HALLMARK_COMPLEMENT
HALLMARK_PI3K_AKT_MTOR_SIGNALING
HALLMARK_BILE_ACID_METABOLISM
HALLMARK_KRAS_SIGNALING_UP
HALLMARK_MYC_TARGETS_V1
HALLMARK_ALLOGRAFT_REJECTION
HALLMARK_APICAL_JUNCTION
HALLMARK_PEROXISOME
HALLMARK_MTORC1_SIGNALING
HALLMARK_P53_PATHWAY
HALLMARK_MITOTIC_SPINDLE
HALLMARK_ESTROGEN_RESPONSE_LATE
HALLMARK_APOPTOSIS
HALLMARK_TNFA_SIGNALING_VIA_NFKB
HALLMARK_WNT_BETA_CATENIN_SIGNALING
B
BM1
EM1
BM2
EM2
KRAS_SIGNALING_DN
SPERMATOGENESIS
HEME_METABOLISM
G2M_CHECKPOINT
E2F_TARGETS
IL6_JAK_STAT3_SIGNALING
INFLAMMATORY_RESPONSE
INTERFERON_ALPHA_RESPONSE
INTERFERON_GAMMA_RESPONSE
PANCREAS_BETA_CELLS
PROTEIN_SECRETION
GLYCOLYSIS
UNFOLDED_PROTEIN_RESPONSE
OXIDATIVE_PHOSPHORYLATION
MYC_TARGETS_V2
MTORC1_SIGNALING
PI3K_AKT_MTOR_SIGNALING
MYC_TARGETS_V1
ALLOGRAFT_REJECTION
DNA_REPAIR
UV_RESPONSE_UP
REACTIVE_OXYGEN_SPECIES_PATHWAY
KRAS_SIGNALING_UP
COMPLEMENT
MITOTIC_SPINDLE
ANDROGEN_RESPONSE
XENOBIOTIC_METABOLISM
APICAL_JUNCTION
P53_PATHWAY
CHOLESTEROL_HOMEOSTASIS
ESTROGEN_RESPONSE_LATE
ESTROGEN_RESPONSE_EARLY
PEROXISOME
FATTY_ACID_METABOLISM
BILE_ACID_METABOLISM
COAGULATION
TGF_BETA_SIGNALING
MYOGENESIS
UV_RESPONSE_DN
EPITHELIAL_MESENCHYMAL_TRANSITION
HEDGEHOG_SIGNALING
NOTCH_SIGNALING
APICAL_SURFACE
WNT_BETA_CATENIN_SIGNALING
APOPTOSIS
HYPOXIA
TNFA_SIGNALING_VIA_NFKB
IL2_STAT5_SIGNALING
ANGIOGENESIS
ADIPOGENESIS
Unsupervised Clusters
1 2 3
C
BM6
BM7
BM8
BM9
Inflammation Class
Low
Medium Low
Medium High
High
D
****
Inflammation Score
0.8
0.7
0.6
BM
EM
Sample
E
****
****
Lee's L Statistic
20
10
0
BM
EM
Visium
Inflammation Score
Opal
IL6
F
BM6
BM7
BM8
BM9
Exhausted
CD8 Dysfunction
Senescence
Treg
G
BM2
EM2
Exhausted
CD8 Dysfunction
Senescence
Treg
Inflammation Class
High
Medium−High
Medium−L
Low
Spot Percent
20%
40%
60%
80%
100%
Z-Score
−2
2

Figure S4: Inferred pathway analysis and relationship with inflammation in acute myeloid leukemia bone marrow and extramedullary tissues. (A) Heatmap of pathway activities in the bone marrow samples BM1, and BM2, and extramedullary samples EM1, and EM2 samples. Hierarchical clustering shows that samples from the same patient shared similar signals. (**B)** Spatial clustering of pathway activities in BM1, EM1, BM2, and EM2. Unsupervised clusters and corresponding pathway profiles are shown, indicating the similarity between cluster 3 of BM1 and the leukemic population cluster of EM1. (**C**) Spatial map of composite inflammation class in validation cohort samples BM6, BM7, BM8, and BM9 (**D**) Comparison of the high-inflammatory class of BM1 and EM1 reveals higher inflammatory activity in the EM environment. **(E)** Lee's L spatial autocorrelation calculation shows significantly positive correlation in BM and EM samples between Visium-based composite inflammatory score and Opal IL-6 protein intensities. (**F**) Localization of T-cell subtypes (exhausted, CD8+ dysfunction, senescence, regulatory T cells [Treg]) based on inflammation class in validation cohort. (**G**) T-cell subtype signatures based on inflammation class in PT2 samples.

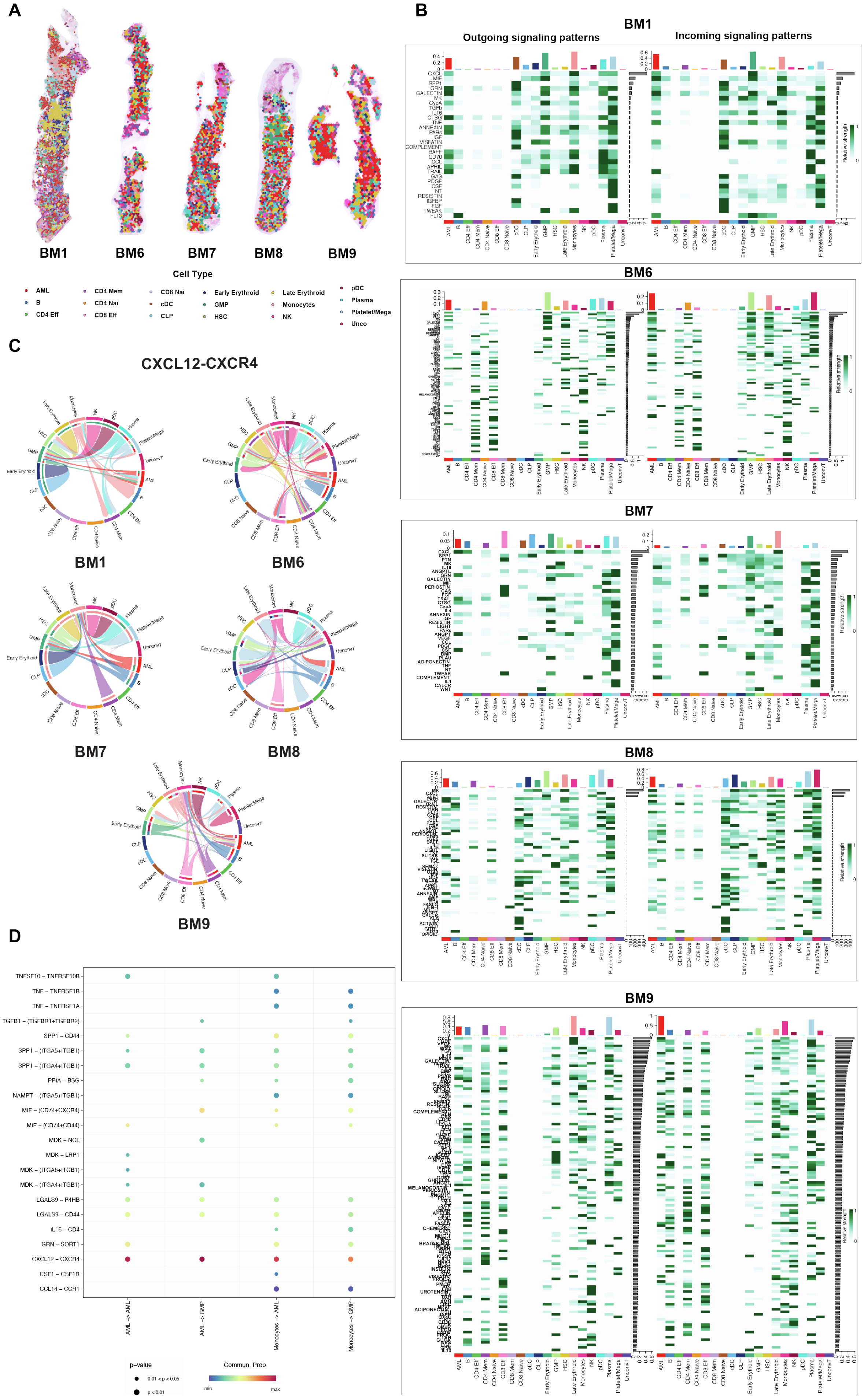

A
BM1
BM6
BM7
BM8
BM9
Cell Type
AML
B
CD4 Eff
CD4 Mem
CD4 Nai
CD8 Eff
CD8 Nai
cDC
CLP
Early Erythroid
GMP
HSC
Late Erythroid
Monocytes
NK
pDC
Plasma
Platelet/Mega
Unco
B
BM1
Outgoing signaling patterns
Incoming signaling patterns
Relative strength
BM6
BM7
BM8
BM9
C
CXCL12-CXCR4
BM1
BM6
BM7
BM8
BM9
D
TNFSF10 – TNFRSF10B
TNF – TNFRSF1B
TNF – TNFRSF1A
TGFB1 – (TGFBR1+TGFBR2)
SPP1 – CD44
SPP1 – (ITGA5+ITGB1)
SPP1 – (ITGA4+ITGB1)
PPIA – BSG
NAMPT – (ITGA5+ITGB1)
MIF – (CD74+CXCR4)
MIF – (CD74+CD44)
MDK – NCL
MDK – LRP1
MDK – (ITGA6+ITGB1)
MDK – (ITGA4+ITGB1)
LGALS9 – P4HB
LGALS9 – CD44
IL16 – CD4
GRN – SORT1
CXCL12 – CXCR4
CSF1 – CSF1R
CCL14 – CCR1
AML -> AML
AML -> GMP
Monocytes -> AML
Monocytes -> GMP
p–value
0.01 < p < 0.05
p < 0.01
Commun. Prob.
min
max

Figure S5: Spatial cell-cell interactions analysis in AML infiltrated bone marrow samples. (A) Spatial map of the bone marrow samples. Spots are labeled by cell type based on deconvolution results, highlighting the distribution of different cell populations. AML, acute myeloid leukemia; Eff, effector T cells; Mem, memory T cells; Nai, naïve T cells; cDC, classical dendritic cells; CLP, common lymphoid progenitors; Early E, early erythroid cells; GMP, granulocyte-monocyte progenitors; HSC, hematopoietic stem cells; Late E, late erythroid cells; NK, natural killer cells; pDC, plasmacytoid dendritic cells; Mega, megakaryocytes; Uncon, unconventional T cells. (**B**) Heatmap of outgoing and incoming signaling patterns for various cell types, showing strong signaling activity in CXCL pathways. **(C)** Chord diagram illustrating the interactions between CXCL12 and CXCR4 across different cell types. **(D)** Dot plot of communication probabilities for significant ligand-receptor pairs in BM1. AML cells, GMPs, and monocytes show strong interactions involving CXCL12-CXCR4.

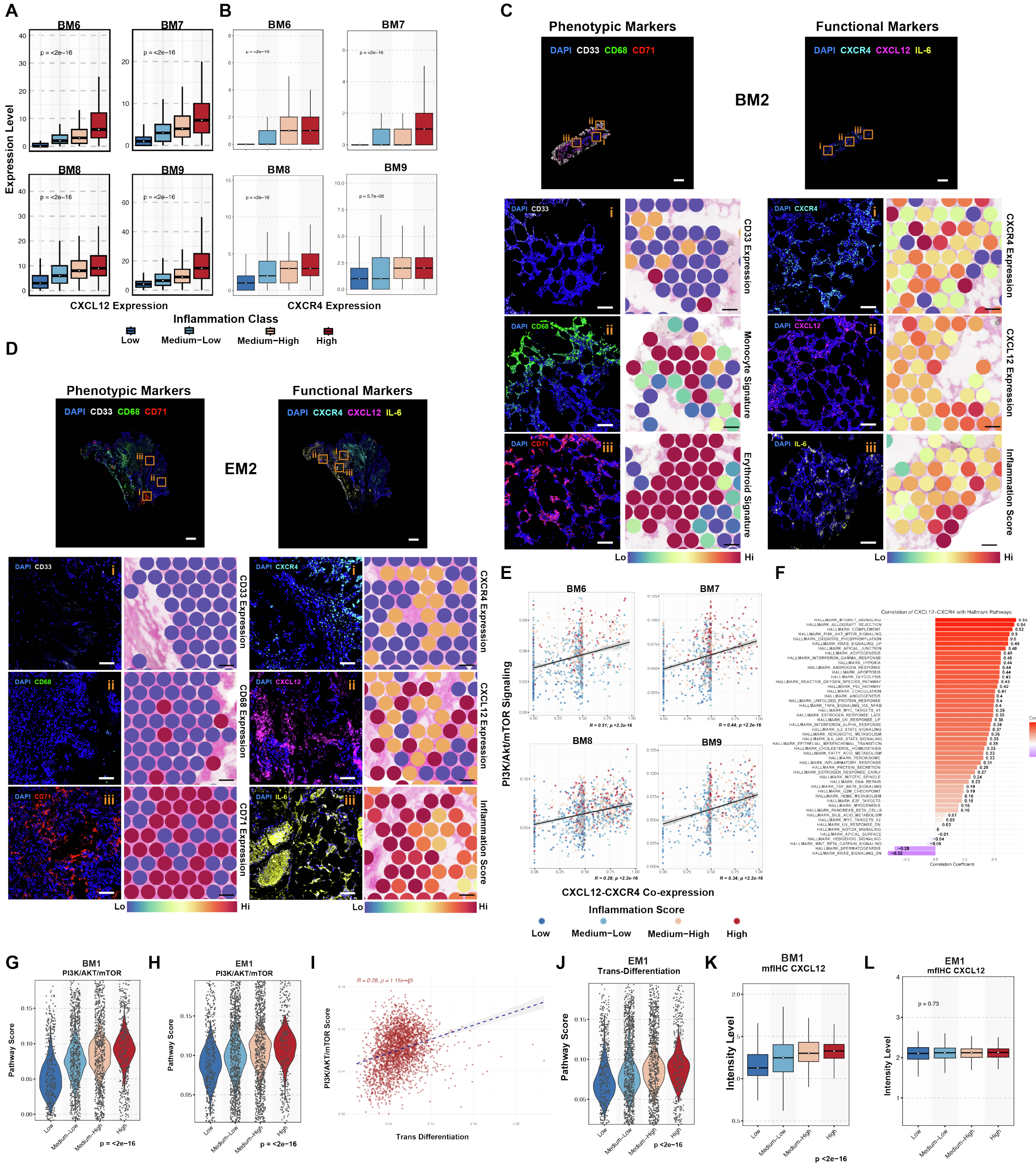

Figure S6: Spatial pathway and cell interaction analysis of the CXCL12-CXCR4 axis in bone marrow and extramedullary tissues from patients with acute myeloid leukemia. (**A**) Boxplots show raw CXCL12 transcript expression across four inflammation classes (Low to High) in BM6–BM9. In all samples, CXCL12 expression was significantly elevated in spots with higher inflammatory activity (Kruskal–Wallis test, $p < 2e{-16}$ for all comparisons). Box center lines represent medians; box limits indicate interquartile range. (**B**) Boxplots display raw CXCR4 transcript expression stratified by inflammation class (Low to High) in the validation cohort. Across all samples, CXCR4 expression was significantly elevated in more inflamed regions (Kruskal–Wallis test; $p < 0.0001$ in all samples). The trend was consistent. **(C and D)** Phenotypic and functional marker staining for the bone marrow sample BM2 (C) and the extramedullary sample EM2 (D) shows strong spatial correlation of multiplex fluorescent immunohistochemistry (mfIHC) signals and Visium signatures. Scale bars: 1 mm (whole-slide panels) and 100 μm (selected region panels).

(**E**) Scatter plots depict the relationship between CXCL12-CXCR4 co-expression scores and PI3K/Akt/mTOR pathway activity across BM6-BM9. Each point represents a Visium spot, colored by inflammation class. In all samples, linear regression shows a consistent positive association between CXCL12–CXCR4 signaling and PI3K/Akt/mTOR pathway activation. (Pearson correlation, $p < 2.2e-16$). **(F)** Correlation of CXCL signaling with other hallmark pathways, highlighting significant associations with inflammation-related pathways in BM1. Specifically, the mTORC1 and PI3K/AKT/mTOR pathways are highly correlated. (**G)** Violin plot of PI3K/AKT/mTOR signaling activity in BM1, stratified by inflammation score categories, indicating higher pathway activity in regions with elevated inflammation. (Kruskal-Wallis test, $p < 2e-16$). (**H)** Violin plot of PI3K/AKT/mTOR signaling activity in the extramedullary sample EM1, stratified by inflammation score categories. (Kruskal-Wallis test, $p < 2e-16$). **(I)** Scatter plot showing the correlation between the composite inflammation score and epithelial-mesenchymal transition (i.e., trans-differentiation) markers in BM1. ($R = 0.28$; $p = 1.15e-45$). **(J)** Violin plot of trans-differentiation signatures in EM1, stratified by inflammation score categories. (Kruskal-Wallis test, $p < 2e-16$). **(K and L)** Comparison of CXCL12 intensities in inflammatory regions across BM1 (K) (Kruskal-Wallis test, $p < 2e-16$) and EM1 (L) (Kruskal-Wallis test, $p = 0.73$), with enrichment in highly inflammatory regions in BM1.

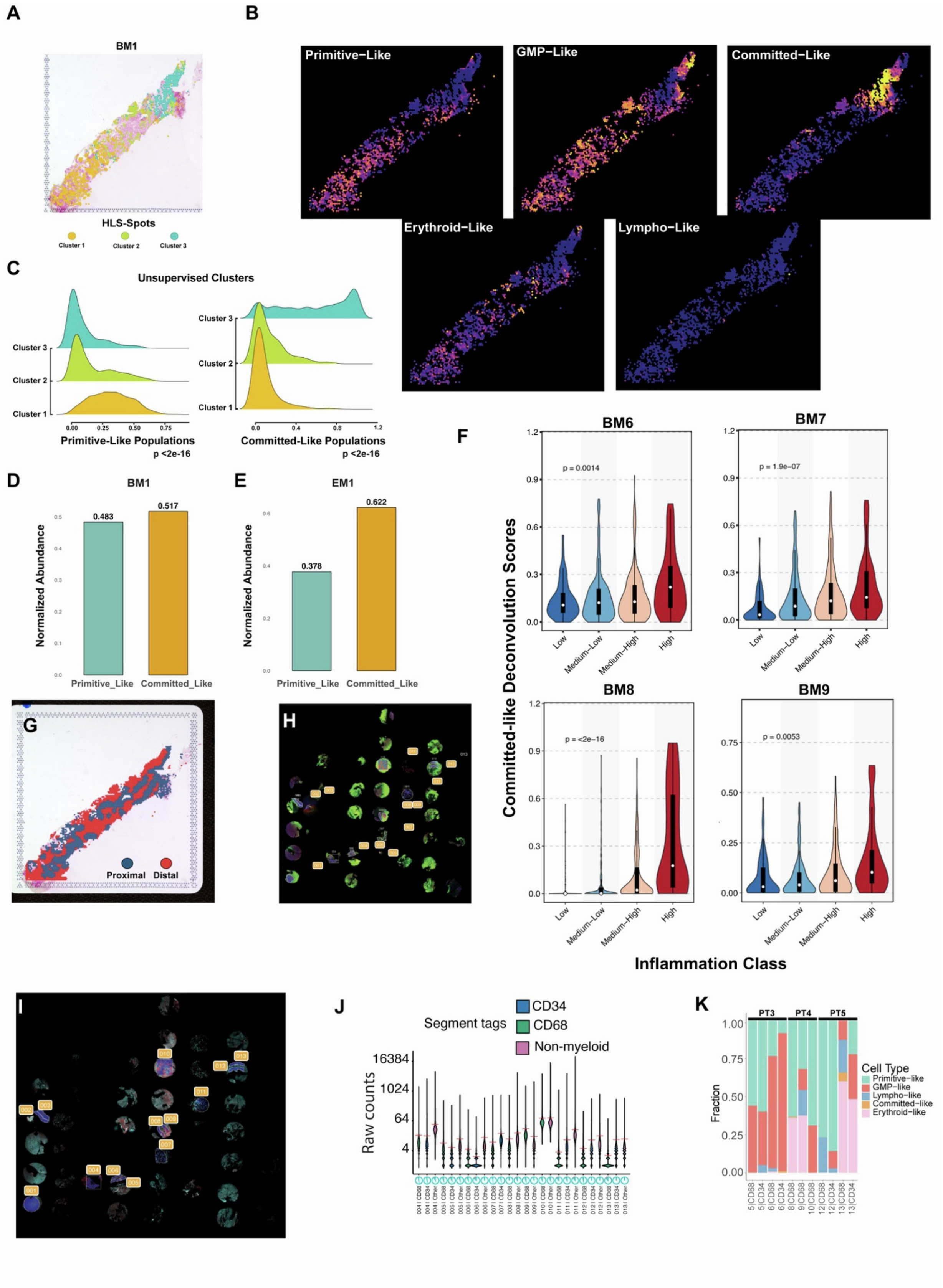
A
BM1
HLS-Spots
Cluster 1
Cluster 2
Cluster 3
B
Primitive−Like
GMP−Like
Committed−Like
Erythroid−Like
Lympho−Like
C
Unsupervised Clusters
Primitive-Like Populations
p <2e-16
Committed-Like Populations
p <2e-16
D
BM1
Normalized Abundance
0.483
0.517
Primitive_Like
Committed_Like
E
EM1
0.378
0.622
F
BM6
p = 0.0014
BM7
p = 1.9e−07
BM8
p = <2e−16
BM9
p = 0.0053
Committed-like Deconvolution Scores
Low
Medium−Low
Medium−High
High
Inflammation Class
G
Proximal
Distal
H
I
J
Segment tags
CD34
CD68
Non-myeloid
Raw counts
K
PT3
PT4
PT5
Fraction
Cell Type
Primitive−like
GMP−like
Lympho−like
Committed−like
Erythroid−like

Figure S7: Classification and Spatial Distribution of AML Cells Based on Differentiation States. **(A)** Spatial map of the bone marrow sample BM1 showing high leukemic score (HLS) spots divided by unsupervised clusters. **(B)** Spatial distribution of different acute myeloid leukemia (AML) cells in BM1, classified as primitive-like, granulocyte-monocyte progenitor (GMP)-like, committed-like, erythroid-like, and lymphoid-like. **(C)** Density plots showing the distribution of primitive-like and committed-like populations across unsupervised clusters in BM1. (Kruskal-Wallis test, $p < 2e-16$)**.** **(D)** Bar graph of deconvolution scores for primitive-like and committed-like cells in BM1, indicating a slightly higher abundance of committed-like cells. **(E)** Bar graph of deconvolution scores for primitive-like and committed-like cells in the extramedullary sample EM1, showing a smaller primitive-like population. (**F**) Violin plots show the distribution of committed-like cell deconvolution scores across inflammation class (Low to High) in validation cohort. In all four samples, committed-like AML populations were significantly enriched in regions with higher inflammatory activity (Kruskal–Wallis test, $p < 0.01$ in all samples). Black bars indicate median and interquartile range. **(G)** Spatial map of BM1 indicating the localization of spots relative to trabecular bone regions. Dark blue indicates proximal; dark red, distal. **(H)** Immunofluorescence image of the GeoMx RNA assay with regions of interest (ROIs) labeled. Areas of illumination (AOIs) captured by GeoMx and segmented by marker expression are shown within each ROI (green, CD34-enriched; red, CD68-enriched; blue, DNA). **(I)** Immunofluorescence image of the GeoMx protein assay with ROI labeled. AOIs captured by GeoMx and segmented by marker expression are shown within each ROI (green, CD34-enriched; red, CD68-enriched; blue, DNA). **(J)** Raw RNA counts of the DNA GeoMx assay. Red lines demark the limit of quantification (LOQ) calculated for each AOI. Pie charts at the bottom show the percentage of genes above the LOQ per segment. **(K)** Deconvolution of GeoMx RNA AOIs using signatures derived from single-cell RNA sequencing data. Primitive-like cells are hematopoietic stem cell–like and common myeloid progenitor/lymphoid-primed multipotent progenitor–like; lymphoid-like cells (Lympho-like) are common lymphoid progenitor–like; and committed-like cells are monocyte-like, basophil-like, and dendritic cell (DC)–like.

Table S1. Patients' characteristics

Characteristic (unit)	PT1	PT2	PT3	PT4	PT5
**Demographics**					
Age (years)	42	83	70	75	74
Sex/gender	Male	Male	Male	Male	Male
Race/ethnicity	Hispanic	Hispanic	White	White	Asian
**Disease progression**					
AML subtype[1]	EM-AML/MS	EM-AML/MS	AMoL/AML-M5	AML-M1	AML-MRC
Origin	De novo	De novo	De novo	De novo	sMDS
Time since diagnosis (months)	0	0	0	0	0
Risk category[2]	Favorable	Adverse	Adverse	Adverse	Adverse
Status	Deceased	Deceased	Deceased	Alive	Deceased
Survival time (months)	2	1	4	67	8
**Spatial Transcriptomic sample information**					
Disease status	Newly Diagnosed	Newly Diagnosed	Newly Diagnosed	Newly Diagnosed	Newly Diagnosed
Collected year	2018	2017	2015	2018	2017
**Data**					
Technology	Visium	Visium	GeoMx	GeoMx	GeoMx
Assay	v1 + v2	v1 + v2	Hu WTA	Hu WTA	Hu WTA
**Cytogenetic and molecular features**					
Karyotype	Diploid	Diploid	Monosomy 7	Diploid	Monosomy 7
Mutations[3]	None detected	DNMT3A, FLT3, IDH1, IDH2, KRAS, NPM1, RAS, SF3B1	DNMT3A, IDH1	IDH2, NPM1, SRSF2	ASXL1, EZH2, KIT, NRAS, RUNX1
**Bone marrow counts**					
Cellularity (%)	40-50	80-90	90-100	90-100	90-100
Myeloblasts (%)	2	30	52	65	31
Monocytes (%)	2	3	17	1	2
**Peripheral blood counts**					
Leukocytes ($\times 10^9$/L)	6.2	11.1	8.3	17.1	25.1
Myeloblasts (%)	0	8	13	80	58
Monocytes (%)	8	6	51	1	7
**Treatment**					
Active agents	CLIA	DAC	DAC	CL+LDAC+VEN / AZA+VEN	CL+LDAC
Response category	NR	NR	NR	CR	NR

Characteristic (unit)	PT6	PT7	PT8	PT9
**Demographics**				
Age (years)	76	70	19	70
Sex/gender	Female	Female	Female	Female
Race/ethnicity	White	Other	White	Black
**Disease progression**				
AML subtype[1]	AML-M1	AML-MRC	AML-M1	AML-MRC
Origin	De novo	Secondary	De novo	Secondary
Time since diagnosis (months)	5	6	33	16
Risk category[2]	Adverse	Adverse	Adverse	Favorable
Status	Deceased	Deceased	Alive	Deceased
Survival time (months)	4	1	65	12
**Spatial Transcriptomic sample information**				
Disease status	Primary Refractory	Relapsed	Relapsed	Relapsed
Collected year	2019	2021	2020	2022
**Data**				
Technology	Visium	Visium	Visium	Visium
Assay	Gene + Protein	Gene + Protein	Gene + Protein	Gene + Protein
**Cytogenetic and molecular features**				
Karyotype	Del7q	Del7q	Complex	Diploid
Mutations[3]	DNMT3A, FLT3, NRAS	SMC3, TET2, TP53	FLT3, NPM1, TP53, WT1	DNMT3A, IDH2, NPM1, WT1
**Bone marrow counts**				
Cellularity (%)	80	10-20	>95	100
Myeloblasts (%)	14	28	65	32
Monocytes (%)	6	1	5	5
**Peripheral blood counts**				
Leukocytes ($\times 10^9$/L)	12.5	2.3	3.6	0.7
Myeloblasts (%)	1	0	8	2
Monocytes (%)	13	4	4	6
**Treatment**				
Active agents	AZA+MDM2 inhibitor	N/A	AZA+VEN+GILT	DAC+VEN+ENA
Response category	NR	N/A	CR	CRi

Abbreviations: EM, extramedullary; MS, myeloid sarcoma; AMoL, acute monocytic leukemia; MRC, myelodysplasia-related changes; sMDS, secondary to myelodysplastic syndrome; v1, Version 1; v2, Version 2; Hu WTA, Human Whole Transcriptome Atlas; CLIA, cladribine, idarubicin, and cytarabine; DAC, decitabine; CL, cladribine; LDAC, low-dose cytarabine; VEN, venetoclax; AZA, azacytidine; NR, no response; CR, complete remission.

[1] AML subtypes were determined according to the World Health Organization's 5th edition[1].
[2] Risk categories were estimated using the European LeukemiaNet 2022 guidelines[2].
[3] Mutations were detected using the 81-gene next-generation sequencing panel, EndLeukemia Assay v1[3].
